# Supplementary figures and images for: Physiological potential and evolutionary trajectories of syntrophic sulfate-reducing bacterial partners of anaerobic methanotrophic archaea
Source: PLoS Biol. 2023 Sep 25;21(9):e3002292. doi: 10.1371/journal.pbio.3002292 (PMC10553843; doi:10.1371/journal.pbio.3002292)

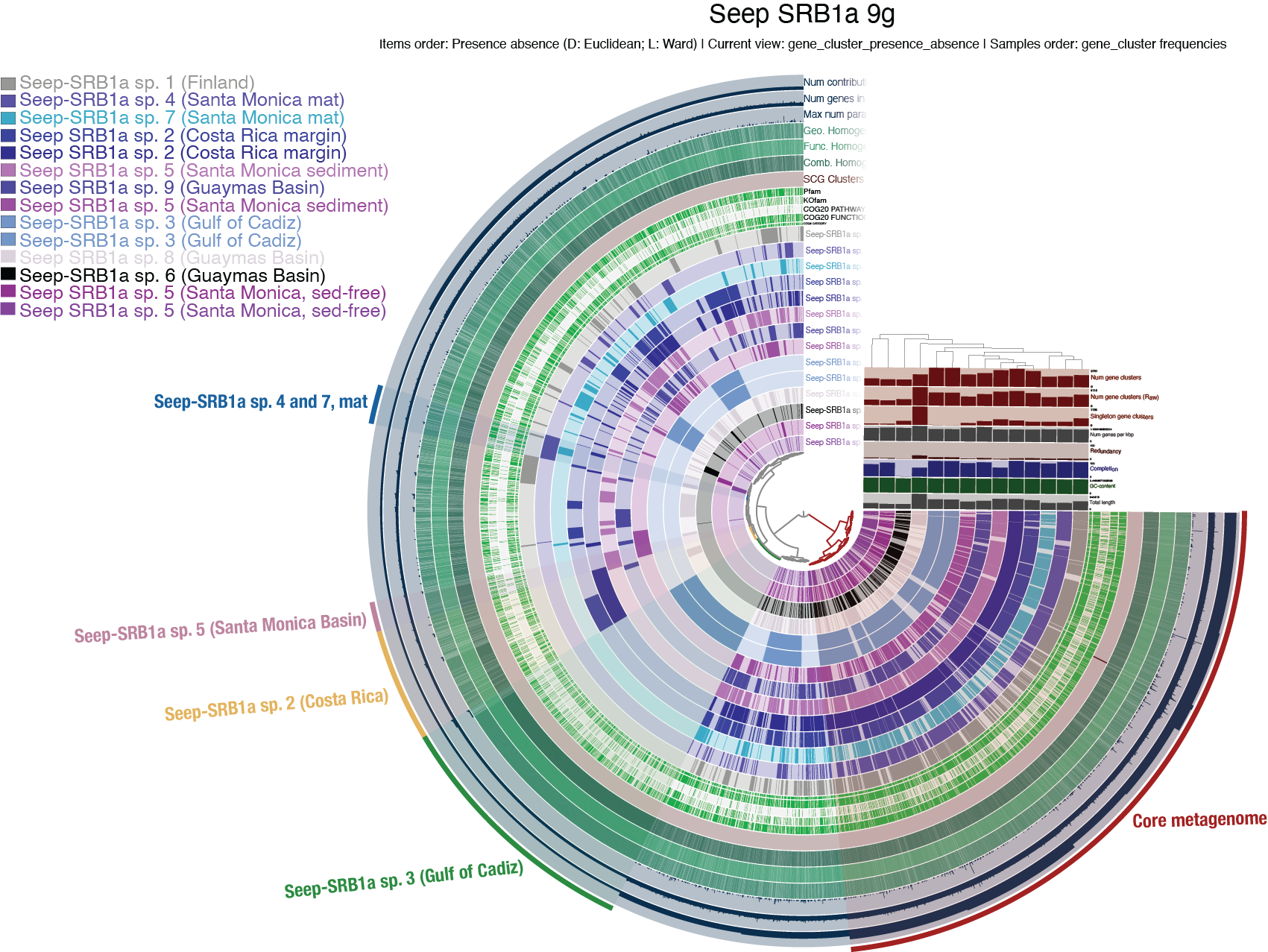

Supplement: S5 Fig — Fourteen genomes from nine Seep-SRB1a species were analyzed using the Anvi’o pan-genome analysis pipeline [45]. Five gene cluster bins were annotated based on genes that were identified as part of the core metagenome, unique to Seep-SRB1a sp. 2, Seep-SRB1a sp. 3, Seep-SRB1a sp. 5, and from the Seep-SRB1a sp. 4 and 7. (PNG) [file pbio.3002292.s005.png]

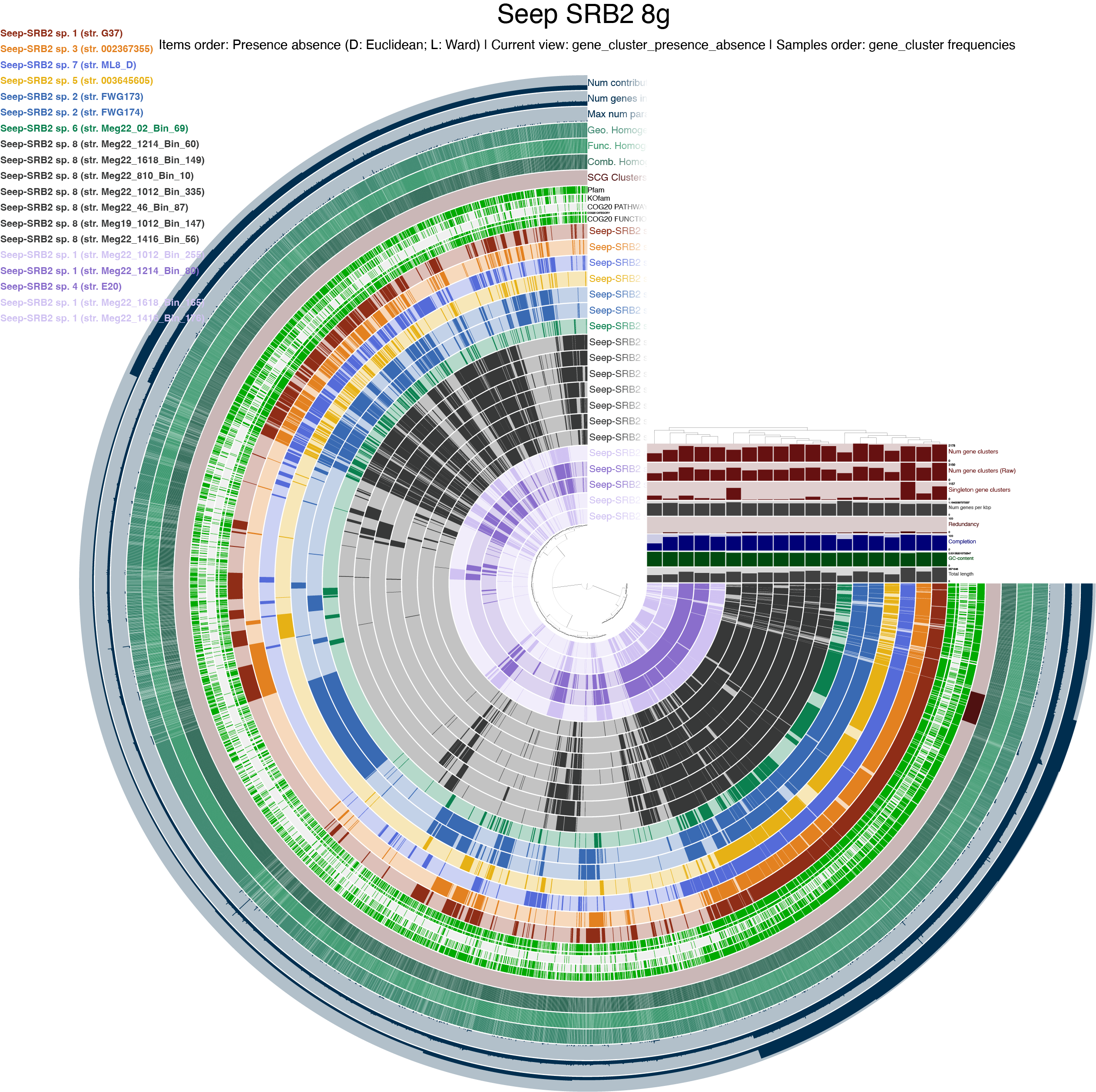

Supplement: S6 Fig — Nineteen genomes from eight Seep-SRB1a species were analyzed using the Anvi’o pan-genome analysis pipeline [45]. Three gene cluster bins were annotated based on genes that were identified as part of the core metagenome, present in Seep-SRB2 sp. 1 and absent in Seep-SRB2 sp. 1. (PNG) [file pbio.3002292.s006.png]

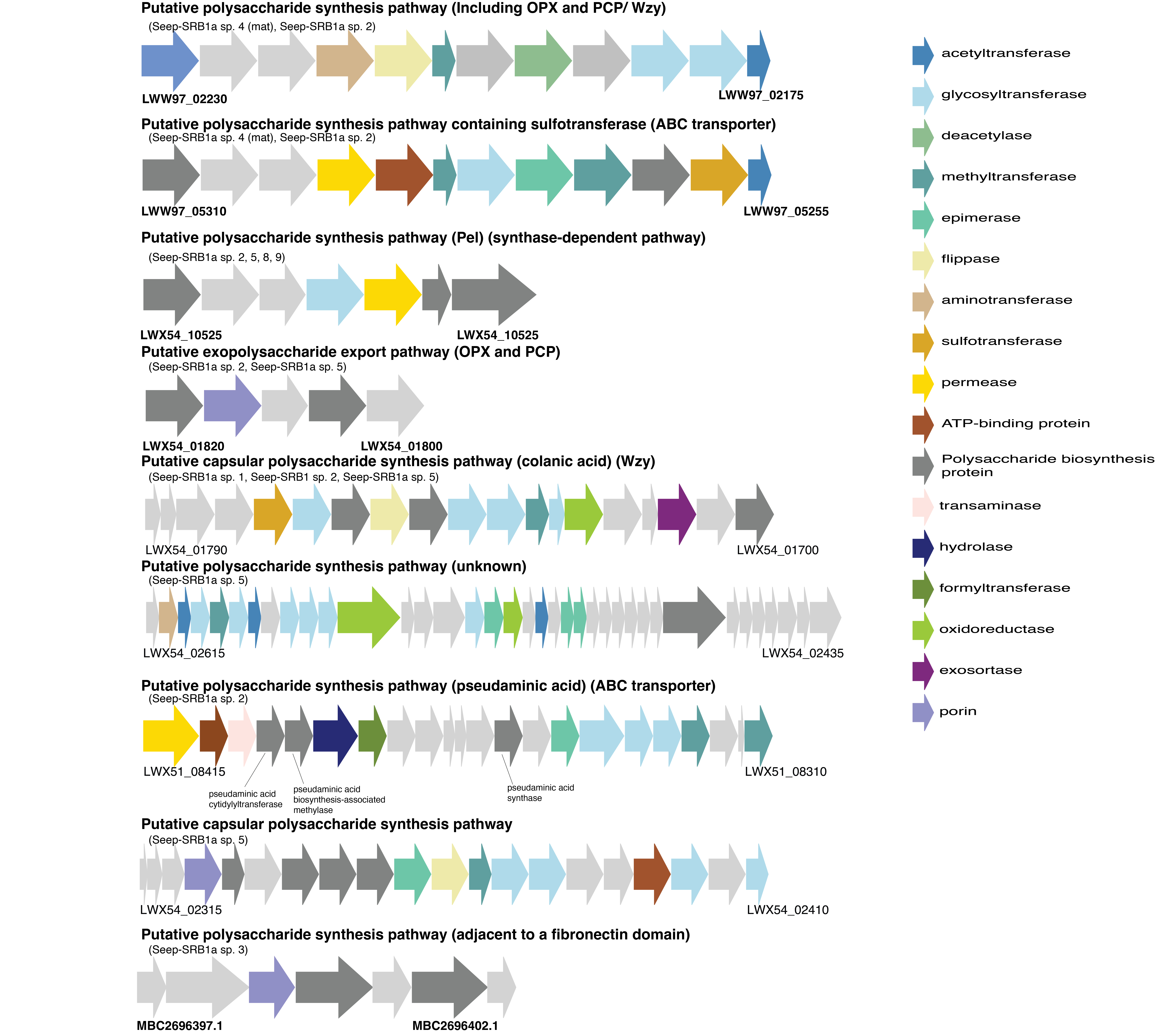

Supplement: S12 Fig — (PNG) [file pbio.3002292.s012.png]

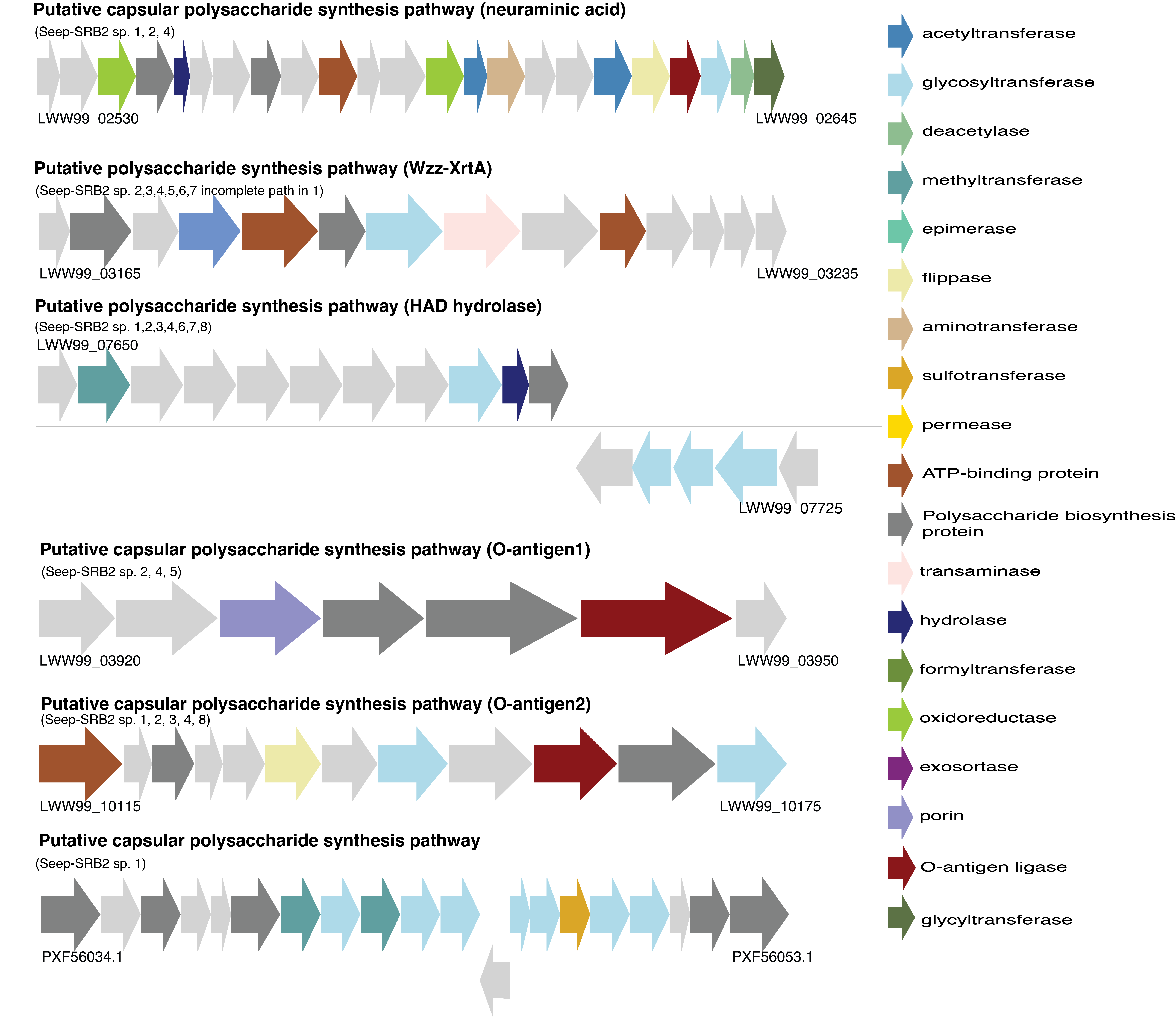

Supplement: S14 Fig — (PNG) [file pbio.3002292.s014.png]

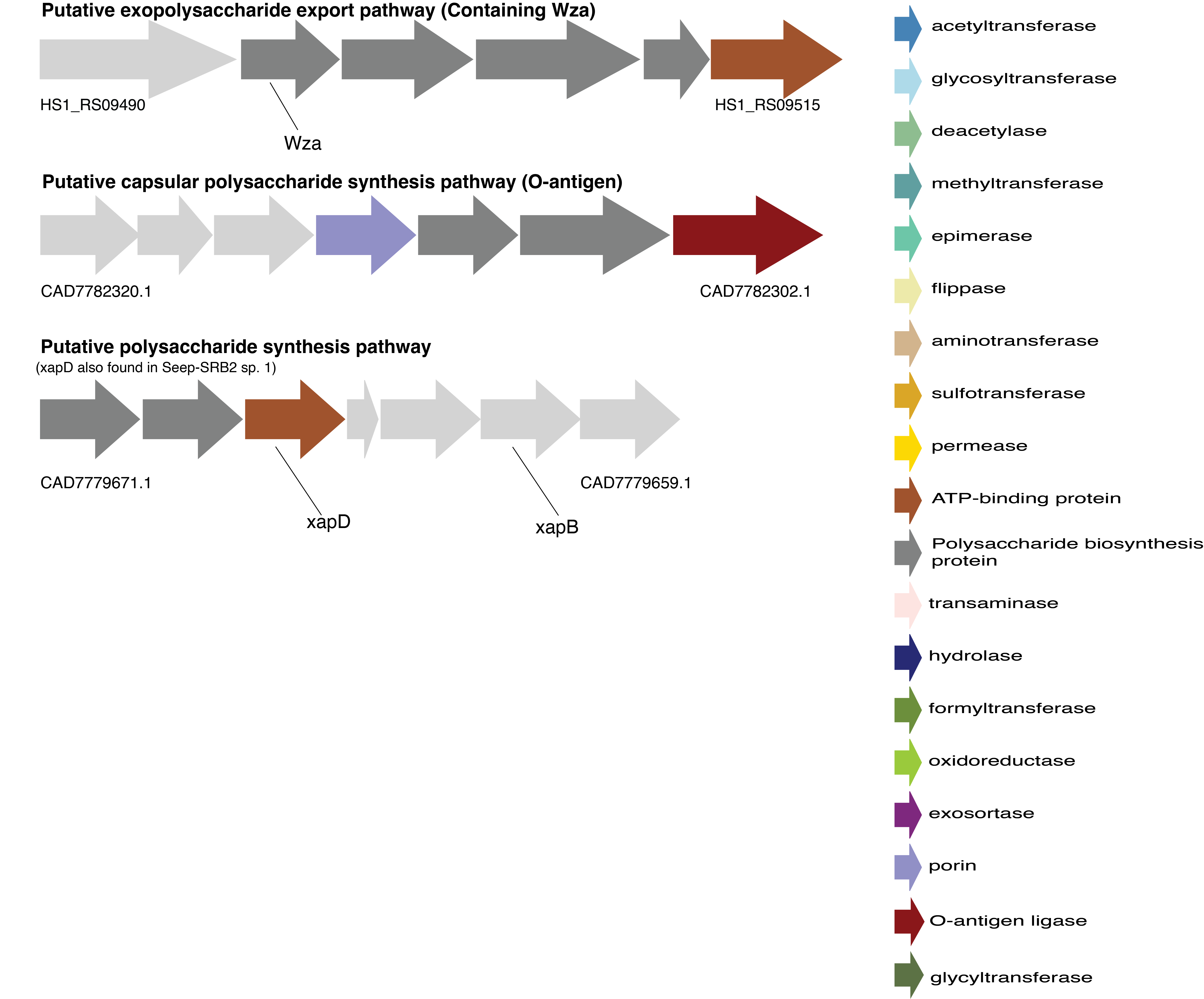

Supplement: S15 Fig — (PNG) [file pbio.3002292.s015.png]

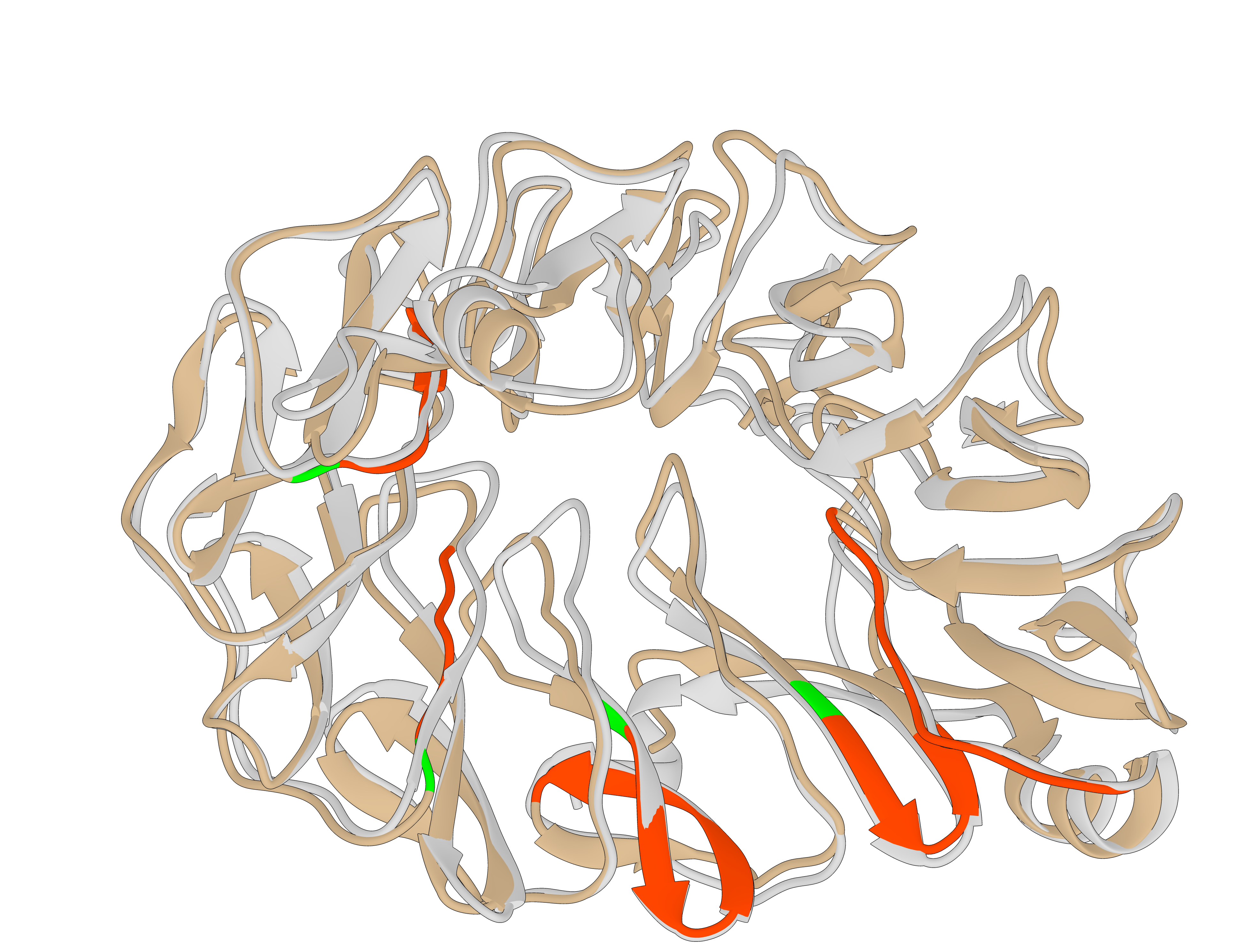

Supplement: S19 Fig — A structural model of TmcD from Seep-SRB1a was generated using Alphafold2 [152] using the monomer option. This model was superimposed on top of a structural model of TmcD from Olavius algarvensis available on UniProt. The divergent sequence regions from TmcD were highlighted in red while cysteine residues unique to Seep-SRB1a were highlighted in green. The conserved residues identified here were observed using a multiple sequence alignment of TmcD made available in online supplementary data. (PNG) [file pbio.3002292.s019.png]

## QmOC tree

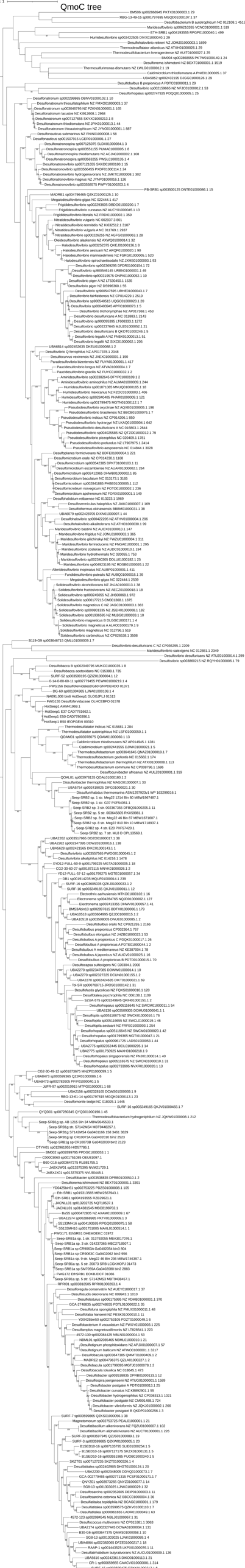

Supplement: S1 Data — Gene_trees_from_syntrophic_SRB.zip. (ZIP) [file pbio.3002292.s035.zip › S1_Data_Gene_trees_from_syntrophic_SRB/QmoC_tree.pdf]

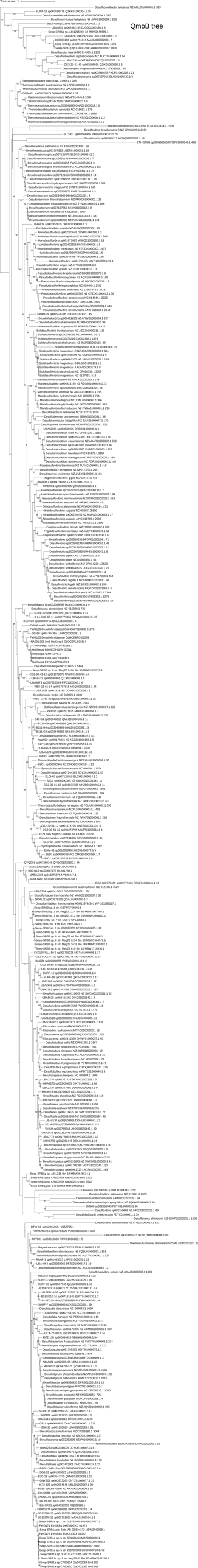

Supplement: S1 Data — Gene_trees_from_syntrophic_SRB.zip. (ZIP) [file pbio.3002292.s035.zip › S1_Data_Gene_trees_from_syntrophic_SRB/QmoB_tree.pdf]

Tree scale: 0.1

# adhesin 8 (dockerin I)

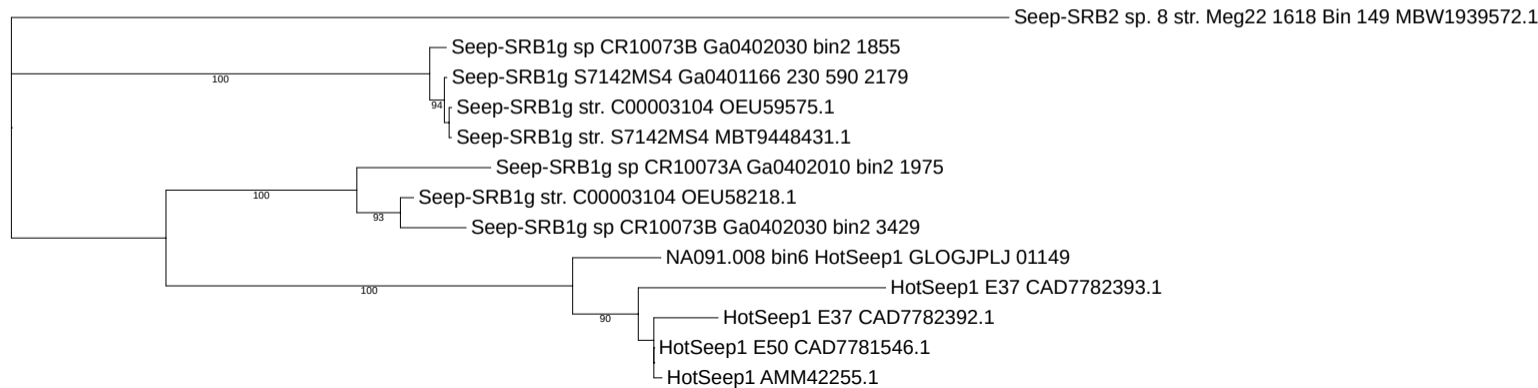

Supplement: S1 Data — Gene_trees_from_syntrophic_SRB.zip. (ZIP) [file pbio.3002292.s035.zip › S1_Data_Gene_trees_from_syntrophic_SRB/adhesin8_dockerinI_iqtree.pdf]

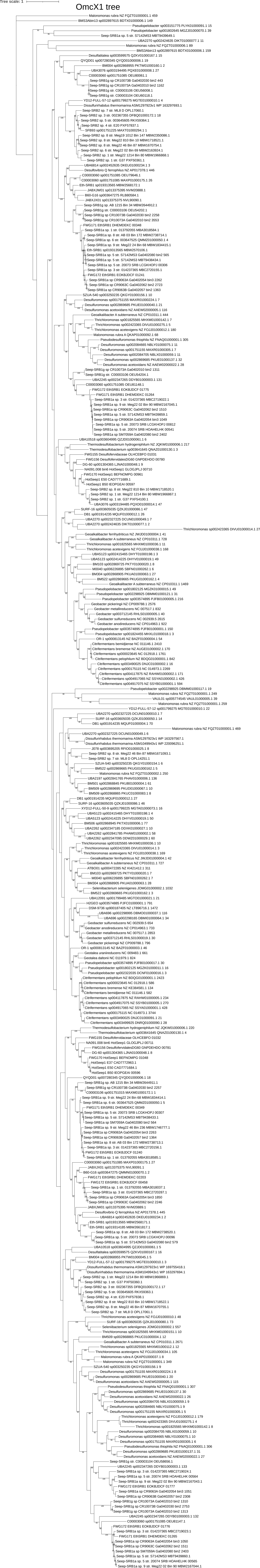

Supplement: S1 Data — Gene_trees_from_syntrophic_SRB.zip. (ZIP) [file pbio.3002292.s035.zip › S1_Data_Gene_trees_from_syntrophic_SRB/OmcX1_tree.pdf]

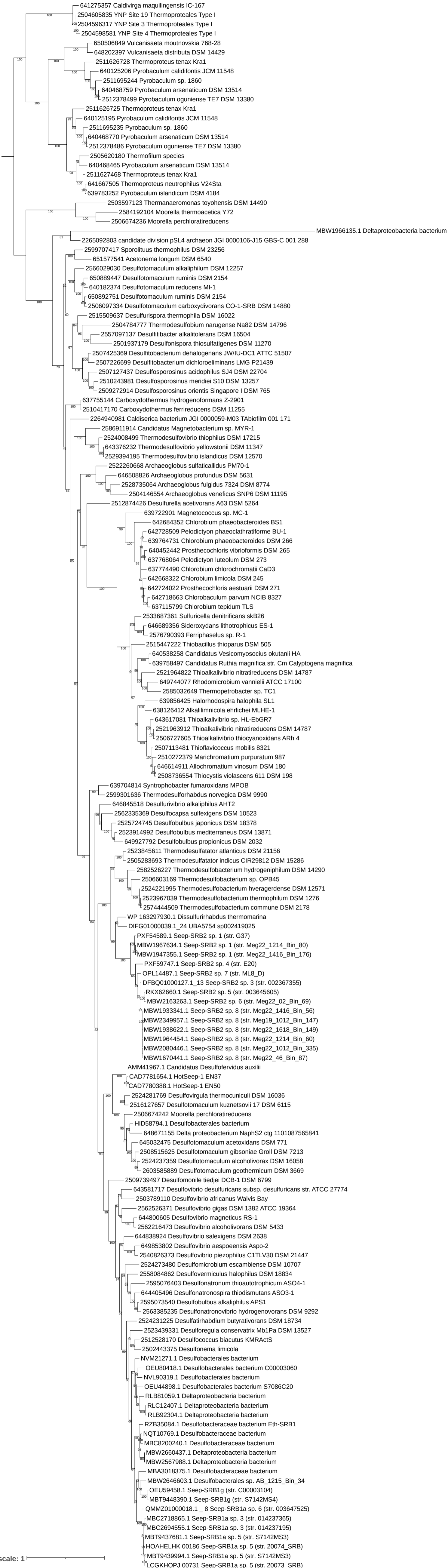

Tree scale: 1

Supplement: S1 Data — Gene_trees_from_syntrophic_SRB.zip. (ZIP) [file pbio.3002292.s035.zip › S1_Data_Gene_trees_from_syntrophic_SRB/DsrB_syntrophic_SRB_against_database.pdf]

Tree scale: 0.1

# integrin

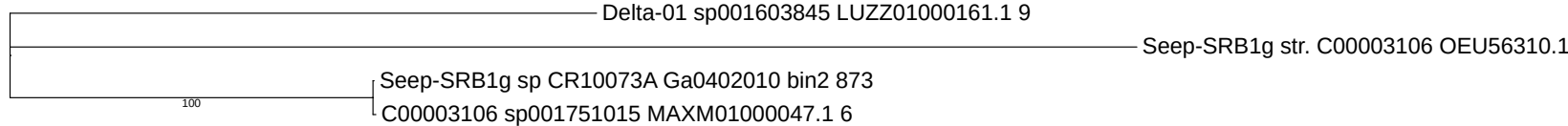

Supplement: S1 Data — Gene_trees_from_syntrophic_SRB.zip. (ZIP) [file pbio.3002292.s035.zip › S1_Data_Gene_trees_from_syntrophic_SRB/adhesin23_integrin_iqtree.pdf]

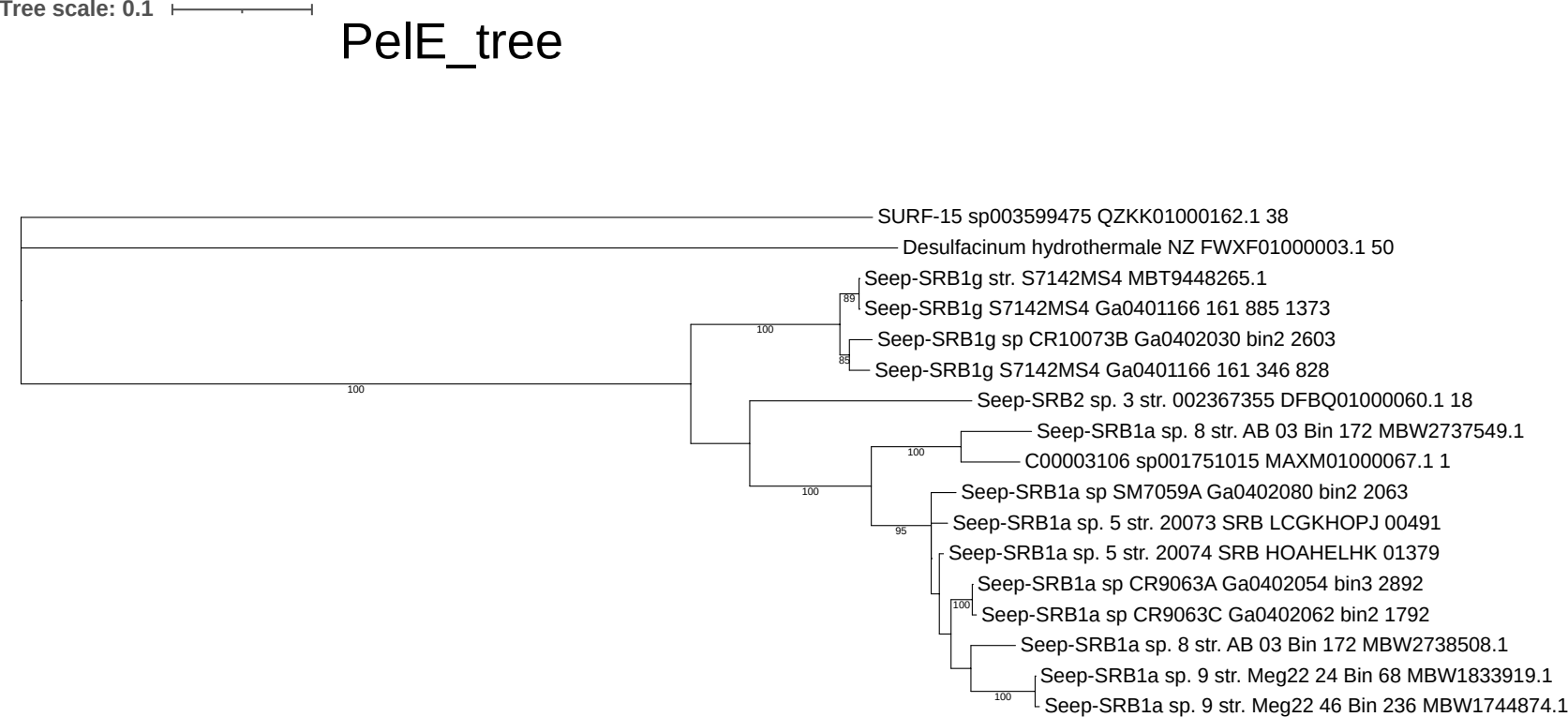

Supplement: S1 Data — Gene_trees_from_syntrophic_SRB.zip. (ZIP) [file pbio.3002292.s035.zip › S1_Data_Gene_trees_from_syntrophic_SRB/PelE_tree.pdf]

# ExtK cytochrome c

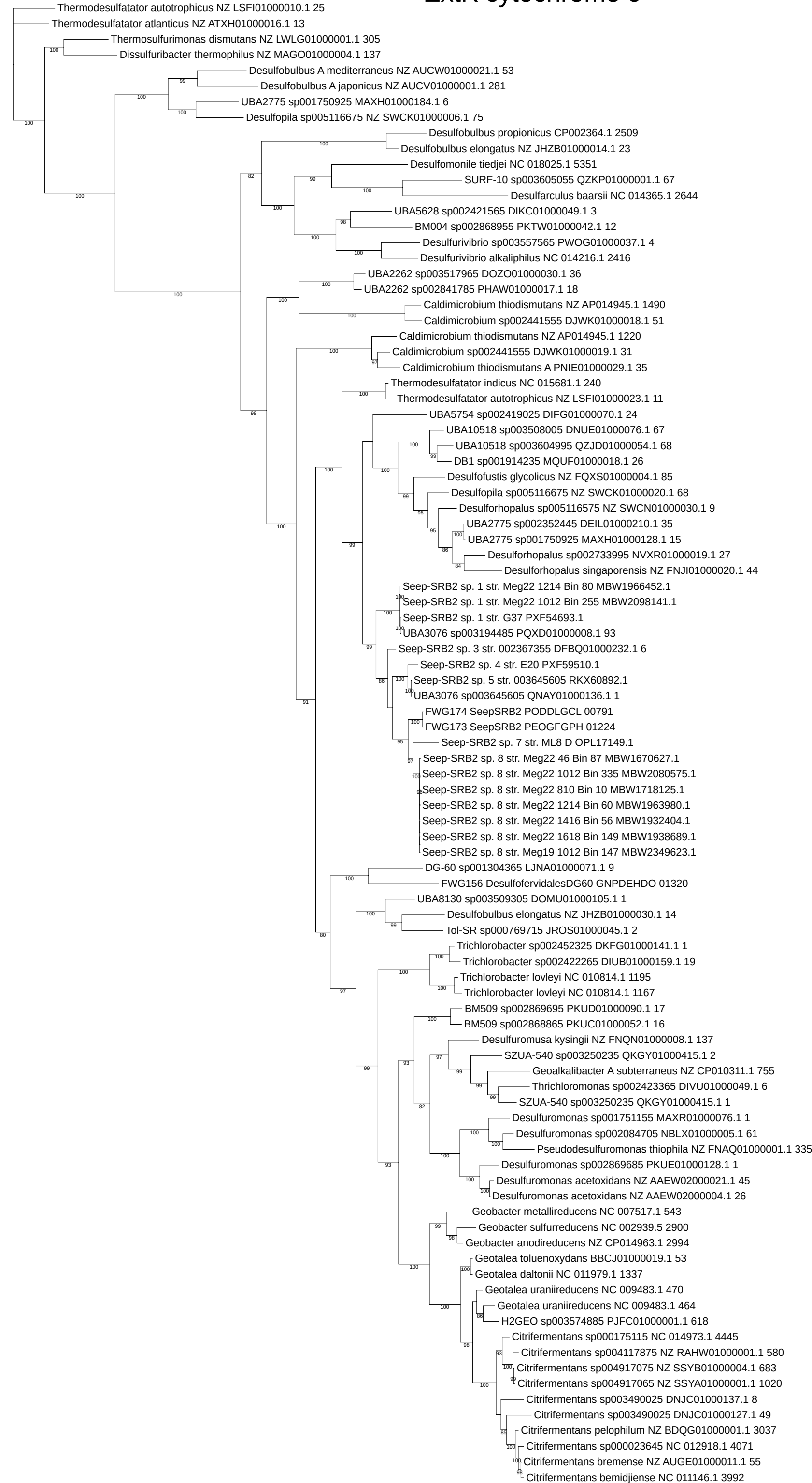

Supplement: S1 Data — Gene_trees_from_syntrophic_SRB.zip. (ZIP) [file pbio.3002292.s035.zip › S1_Data_Gene_trees_from_syntrophic_SRB/extK_cytochrome_c_iqtree.pdf]

Tree scale: 1

# Apc2a

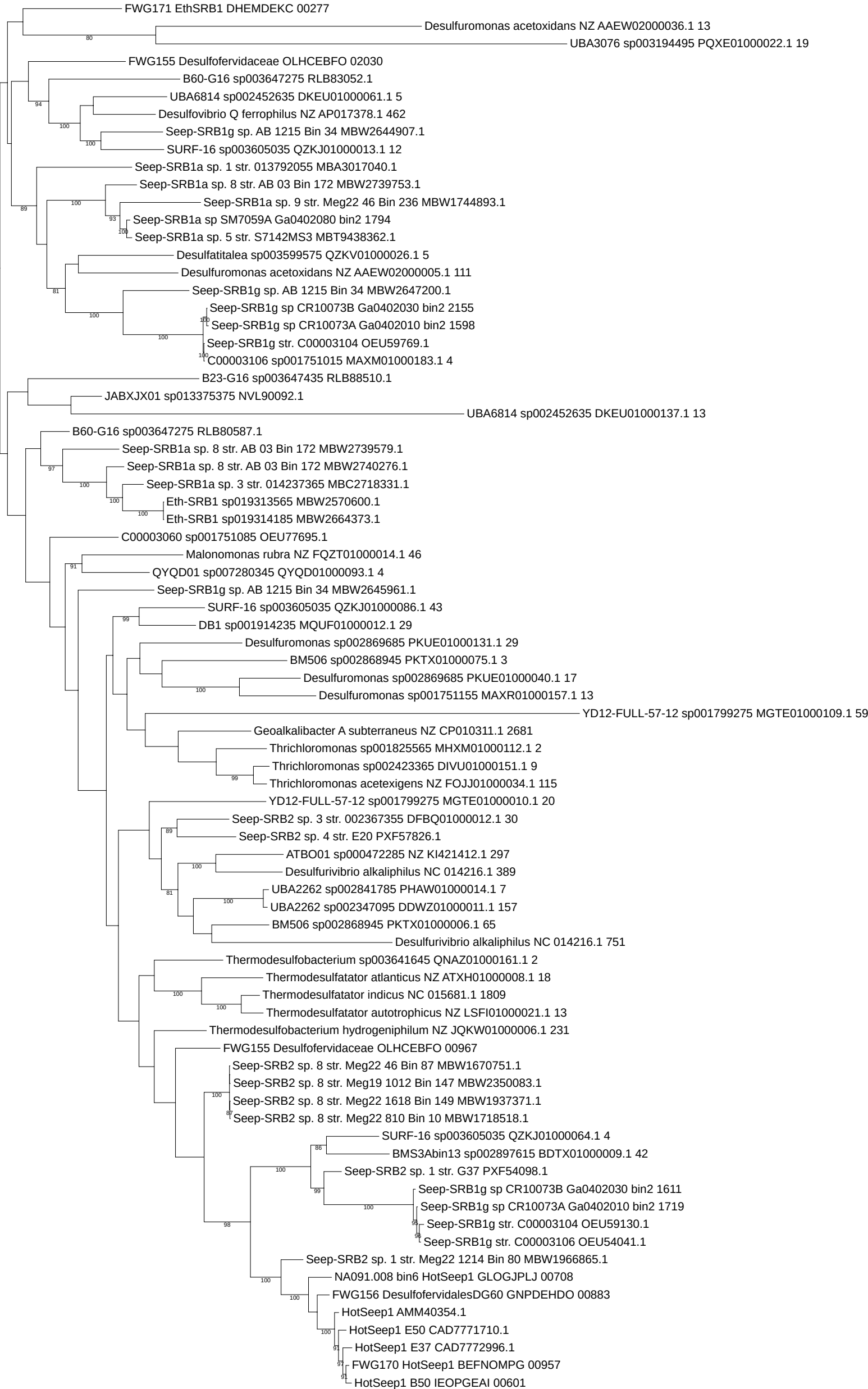

Supplement: S1 Data — Gene_trees_from_syntrophic_SRB.zip. (ZIP) [file pbio.3002292.s035.zip › S1_Data_Gene_trees_from_syntrophic_SRB/Apc2a_tree.pdf]

Tree scale: 0.1

# adhesin 4 (PEGA domain containing protein)

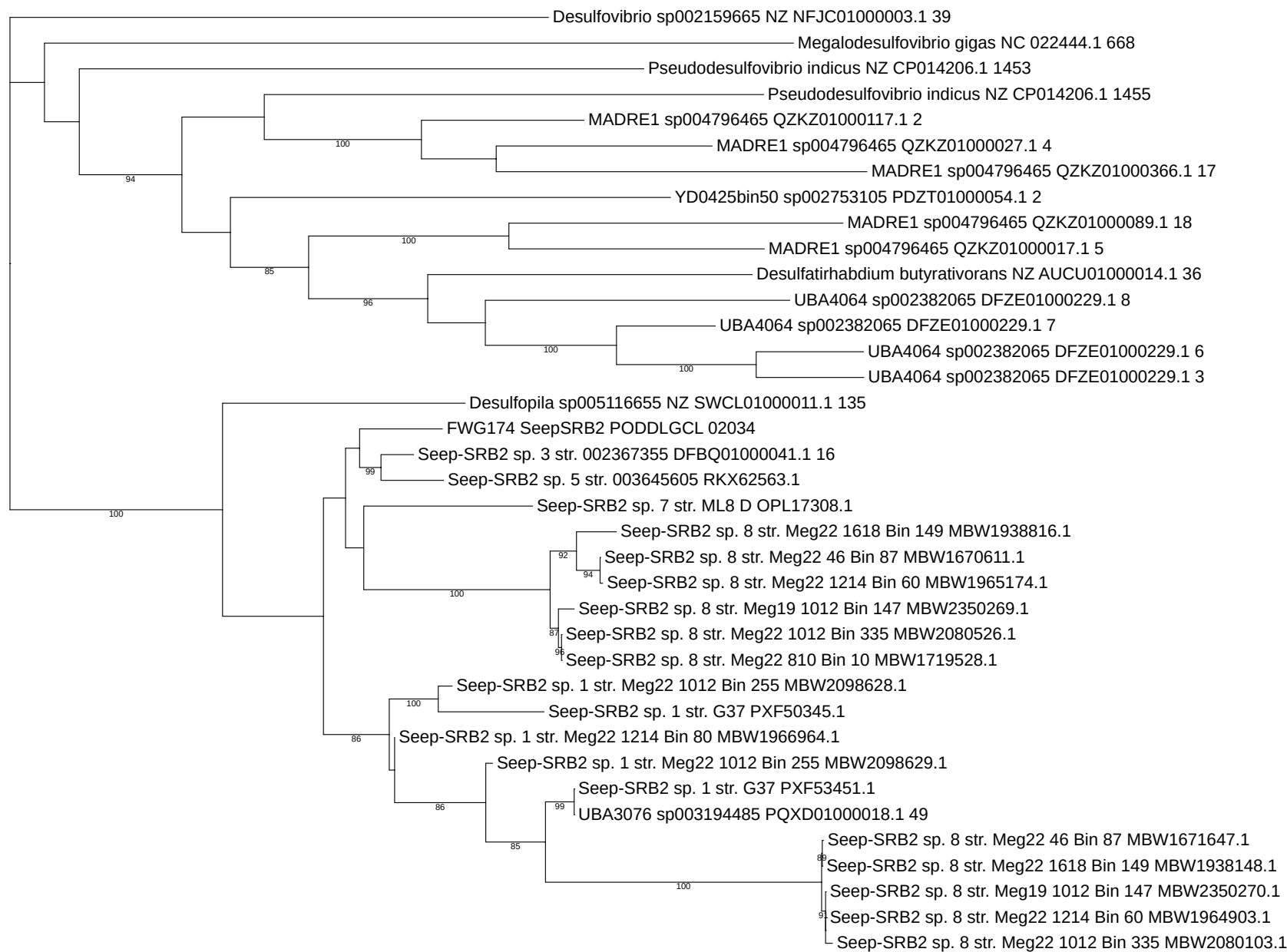

Supplement: S1 Data — Gene_trees_from_syntrophic_SRB.zip. (ZIP) [file pbio.3002292.s035.zip › S1_Data_Gene_trees_from_syntrophic_SRB/adhesin4_PEGA_domain_iqtree.pdf]

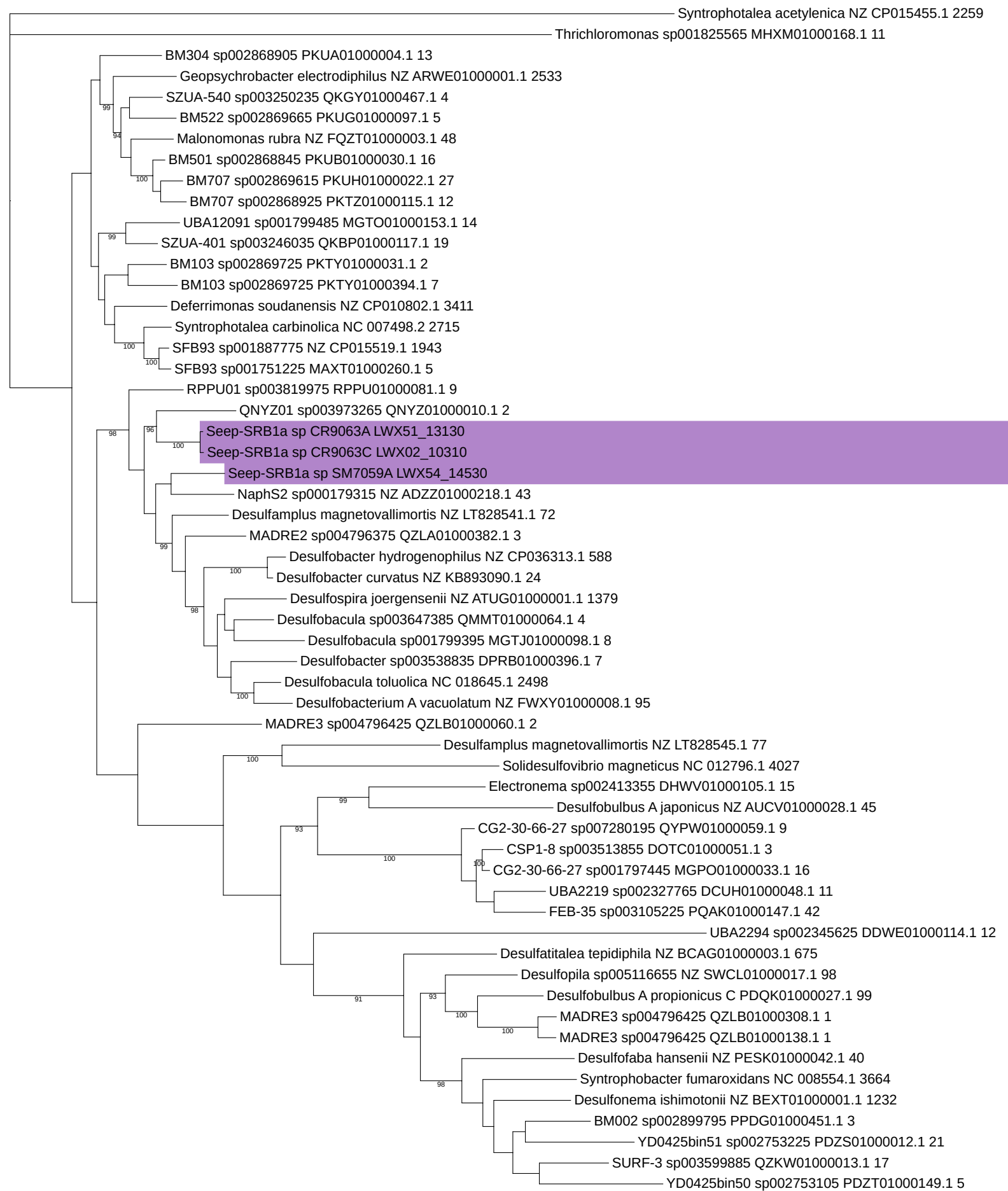

Supplement: S1 Data — Gene_trees_from_syntrophic_SRB.zip. (ZIP) [file pbio.3002292.s035.zip › S1_Data_Gene_trees_from_syntrophic_SRB/adhesin1_vWA_domain_iqtree.pdf]

## Fibronectin\_binding\_domain

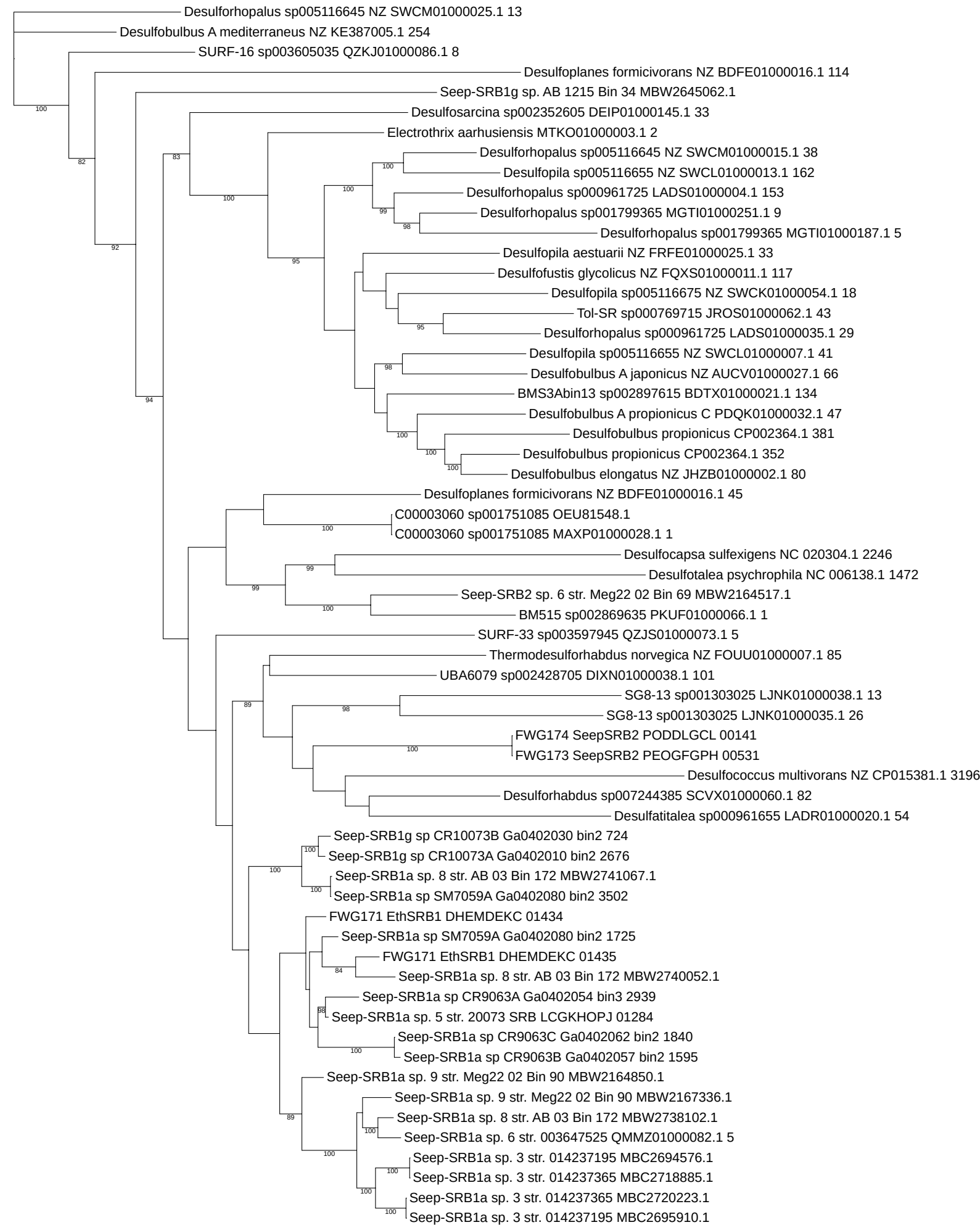

Supplement: S1 Data — Gene_trees_from_syntrophic_SRB.zip. (ZIP) [file pbio.3002292.s035.zip › S1_Data_Gene_trees_from_syntrophic_SRB/adhesin22_fibronectin_binding_domain_iqtree.pdf]

## Pelf tree

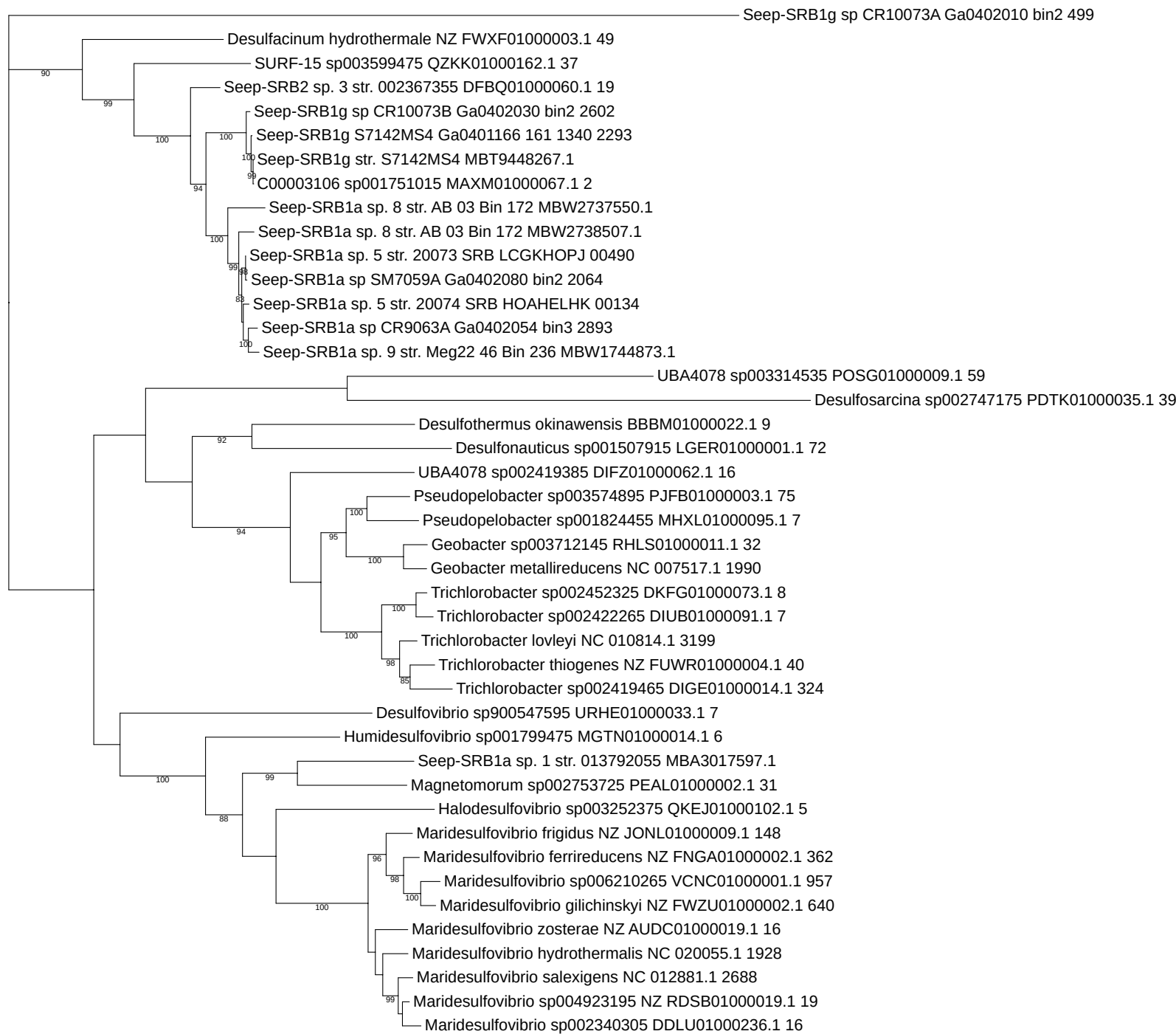

Supplement: S1 Data — Gene_trees_from_syntrophic_SRB.zip. (ZIP) [file pbio.3002292.s035.zip › S1_Data_Gene_trees_from_syntrophic_SRB/PelF_tree.pdf]

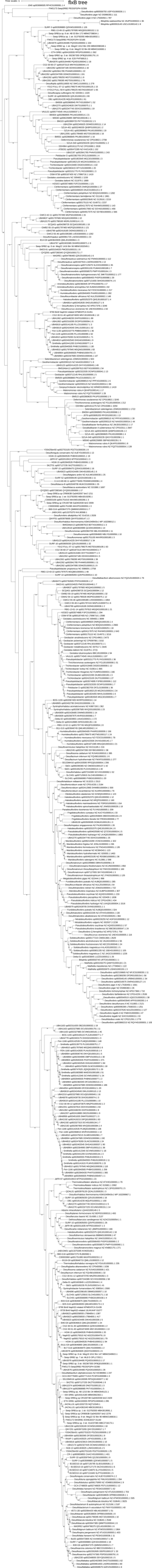

Supplement: S1 Data — Gene_trees_from_syntrophic_SRB.zip. (ZIP) [file pbio.3002292.s035.zip › S1_Data_Gene_trees_from_syntrophic_SRB/flxB_tree.pdf]

## QrcD tree

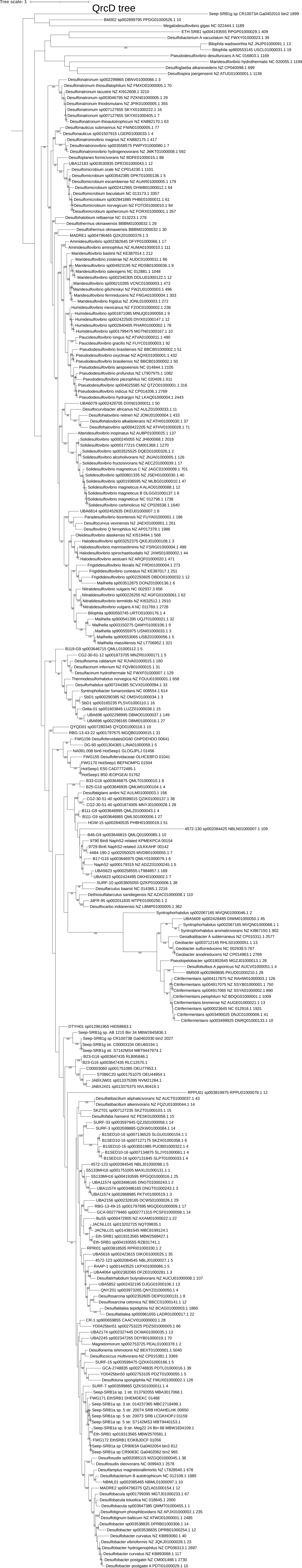

Supplement: S1 Data — Gene_trees_from_syntrophic_SRB.zip. (ZIP) [file pbio.3002292.s035.zip › S1_Data_Gene_trees_from_syntrophic_SRB/QrcD_tree.pdf]

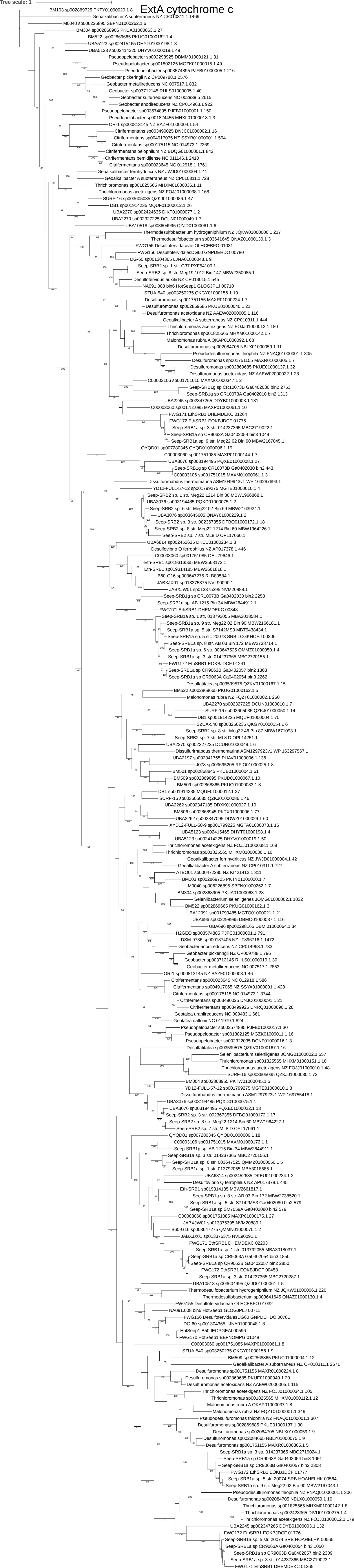

Supplement: S1 Data — Gene_trees_from_syntrophic_SRB.zip. (ZIP) [file pbio.3002292.s035.zip › S1_Data_Gene_trees_from_syntrophic_SRB/ExtA_cytochrome_c_iqtree.pdf]

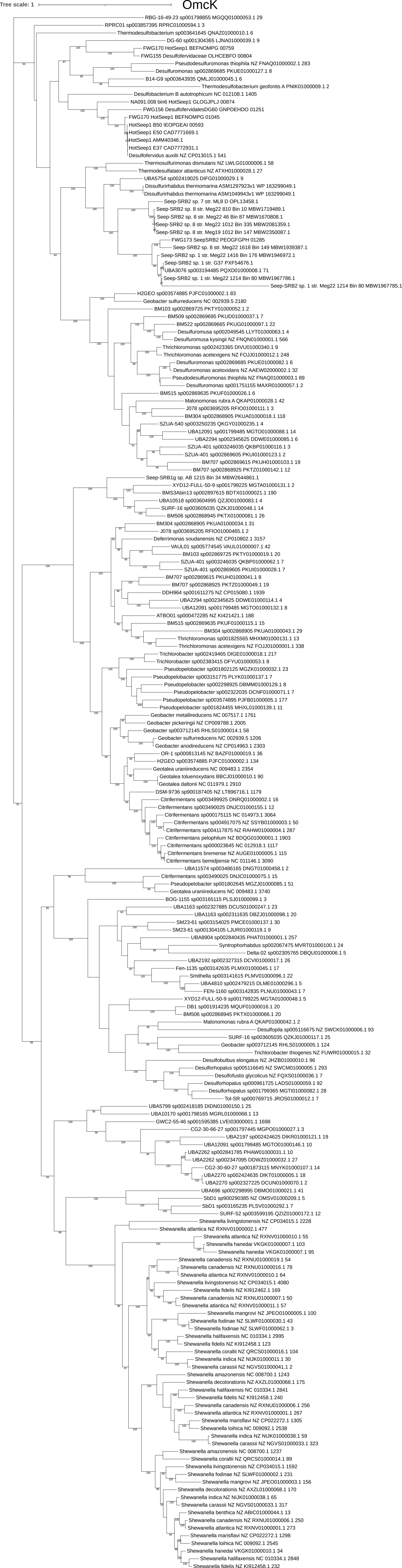

Supplement: S1 Data — Gene_trees_from_syntrophic_SRB.zip. (ZIP) [file pbio.3002292.s035.zip › S1_Data_Gene_trees_from_syntrophic_SRB/OmcK_iqtree.pdf]

## DsrB

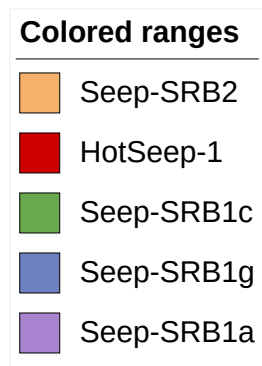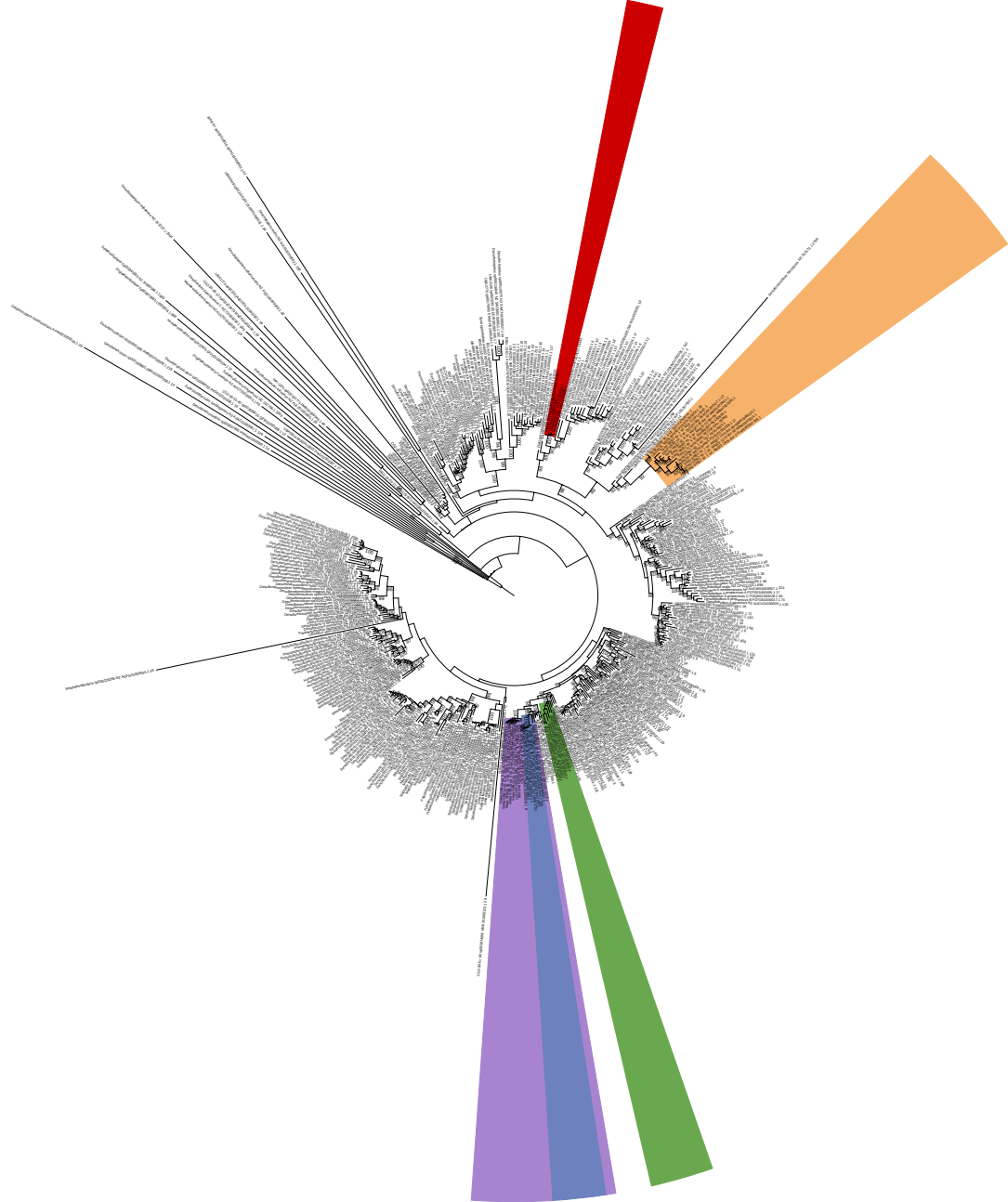

Supplement: S1 Data — Gene_trees_from_syntrophic_SRB.zip. (ZIP) [file pbio.3002292.s035.zip › S1_Data_Gene_trees_from_syntrophic_SRB/DsrB_tree_sulf.pdf]

Tree scale: 1

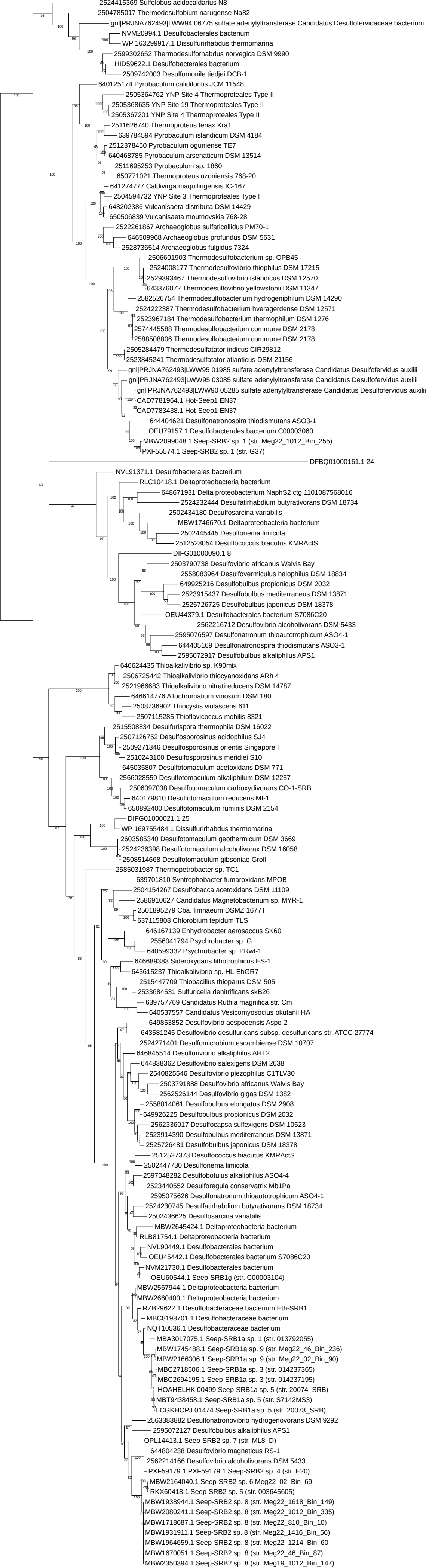

Supplement: S1 Data — Gene_trees_from_syntrophic_SRB.zip. (ZIP) [file pbio.3002292.s035.zip › S1_Data_Gene_trees_from_syntrophic_SRB/SatA_tree_rooted_at_thermophilic_group.pdf]

## OmcL

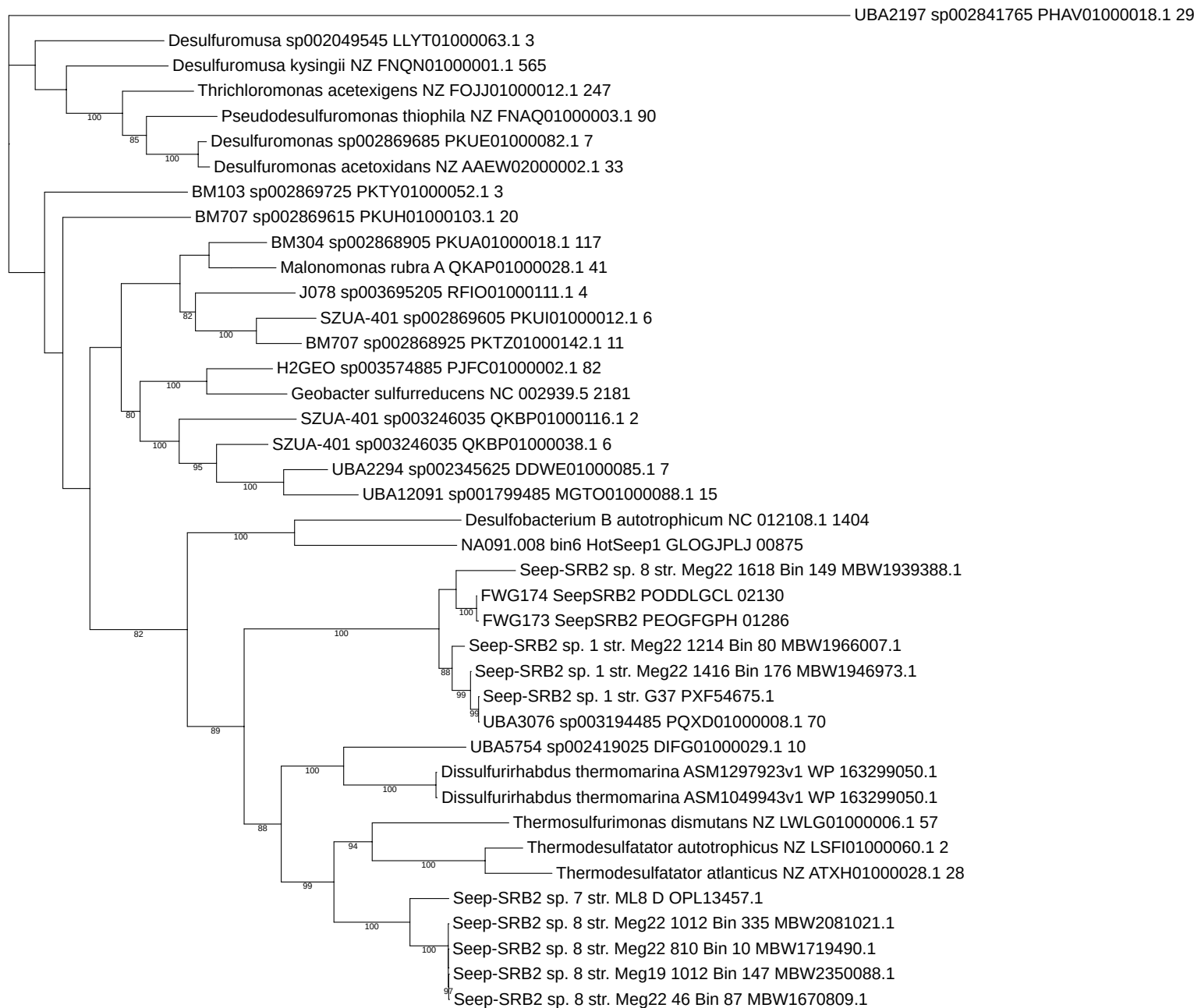

Supplement: S1 Data — Gene_trees_from_syntrophic_SRB.zip. (ZIP) [file pbio.3002292.s035.zip › S1_Data_Gene_trees_from_syntrophic_SRB/OmcL_iqtree.pdf]

# Immunoglobulin-like domain

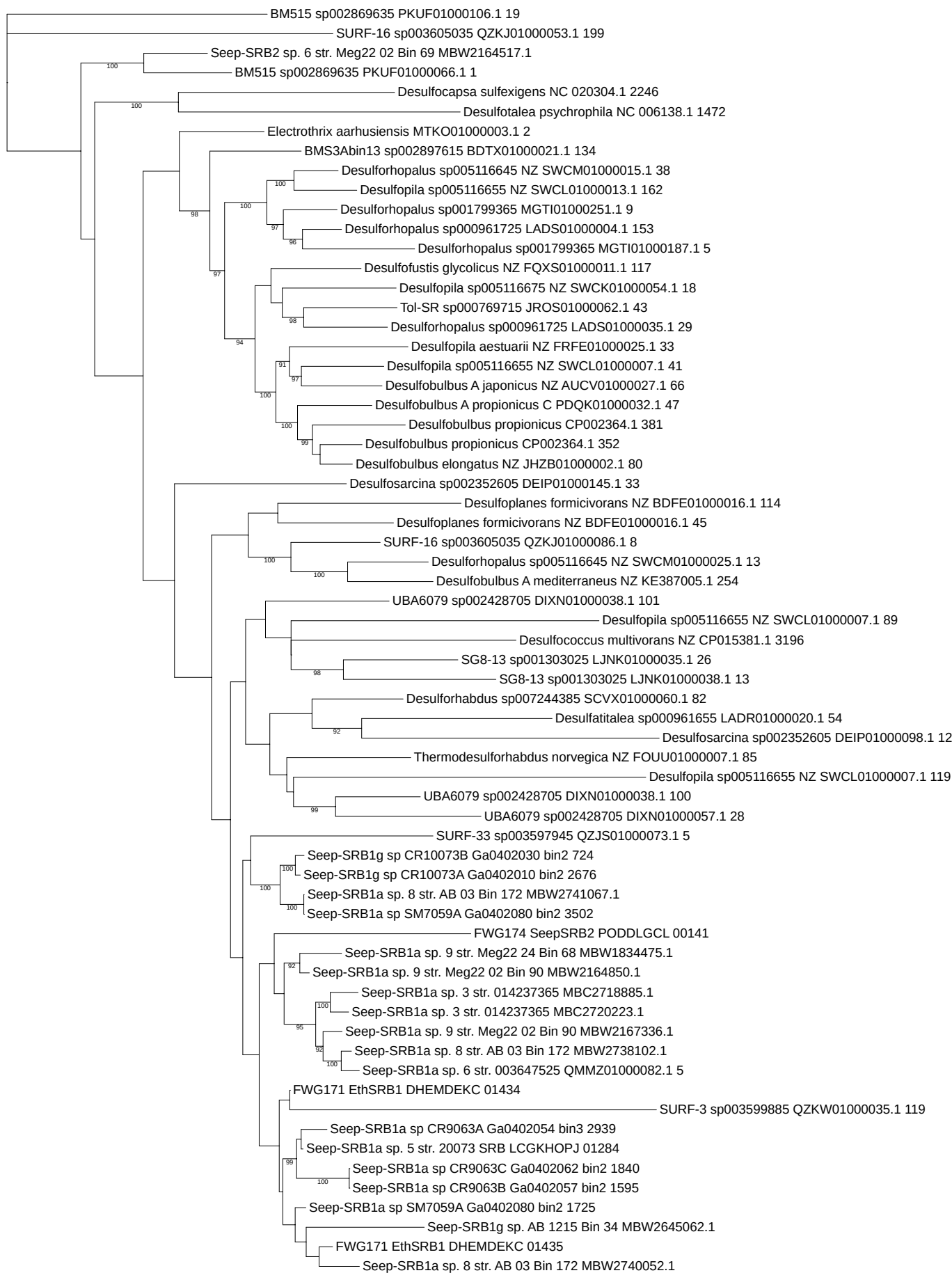

Supplement: S1 Data — Gene_trees_from_syntrophic_SRB.zip. (ZIP) [file pbio.3002292.s035.zip › S1_Data_Gene_trees_from_syntrophic_SRB/adhesin21_Iglike_domain.pdf]

Tree scale: 1

# CARDB domain

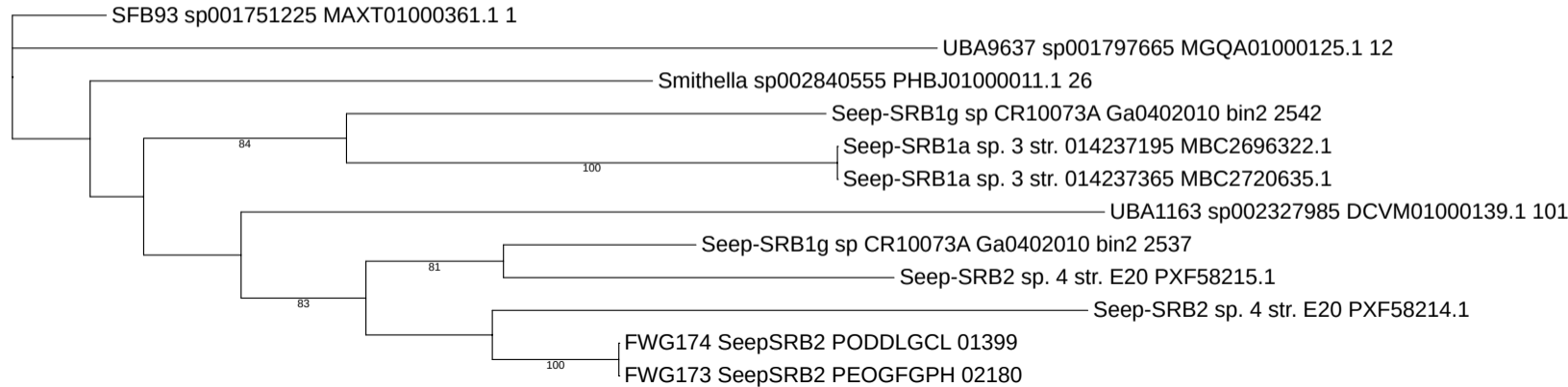

Supplement: S1 Data — Gene_trees_from_syntrophic_SRB.zip. (ZIP) [file pbio.3002292.s035.zip › S1_Data_Gene_trees_from_syntrophic_SRB/adhesin28_CARDB_domain_iqtree.pdf]

Tree scale: 1

DsrA

### Colored ranges

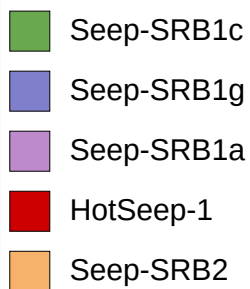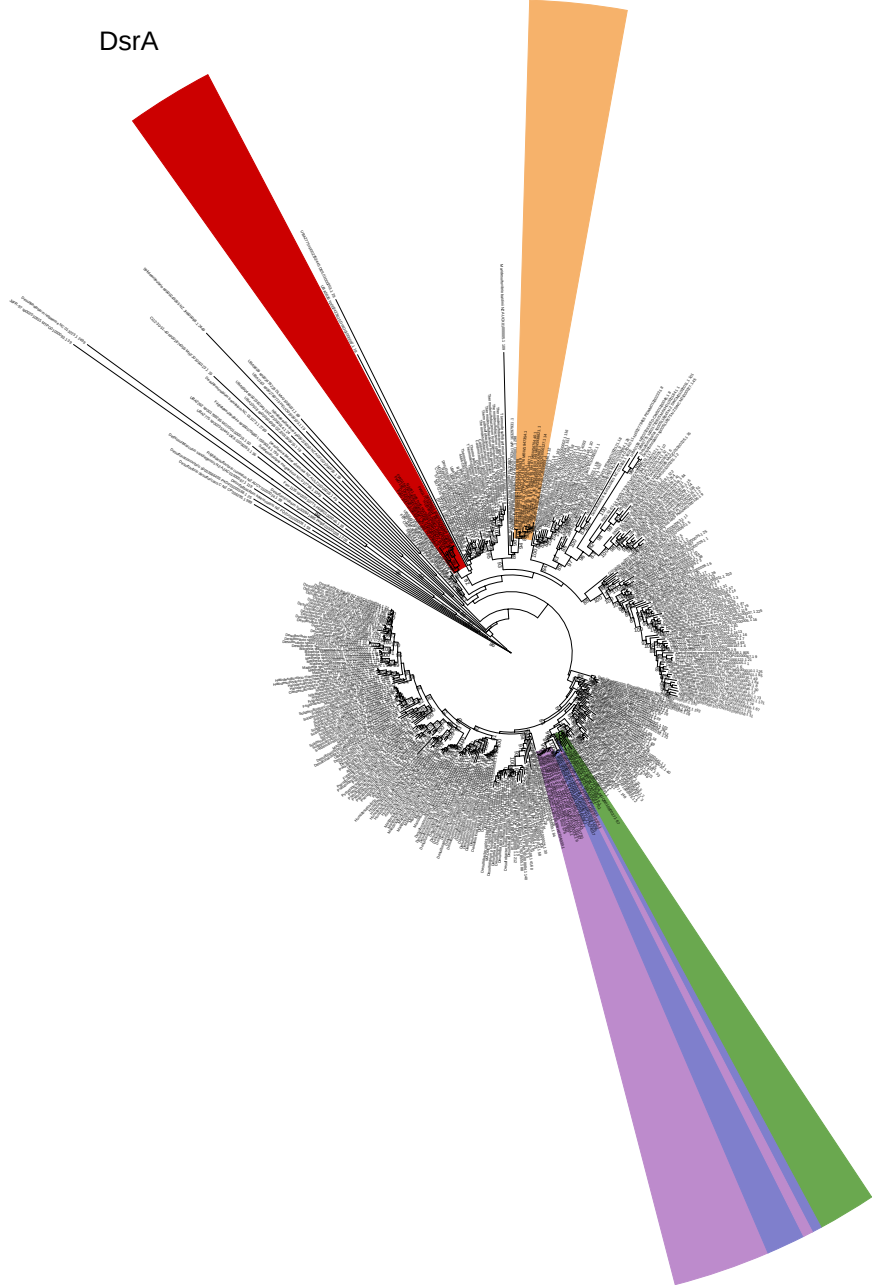

Supplement: S1 Data — Gene_trees_from_syntrophic_SRB.zip. (ZIP) [file pbio.3002292.s035.zip › S1_Data_Gene_trees_from_syntrophic_SRB/DsrA_tree_Desulf.pdf]

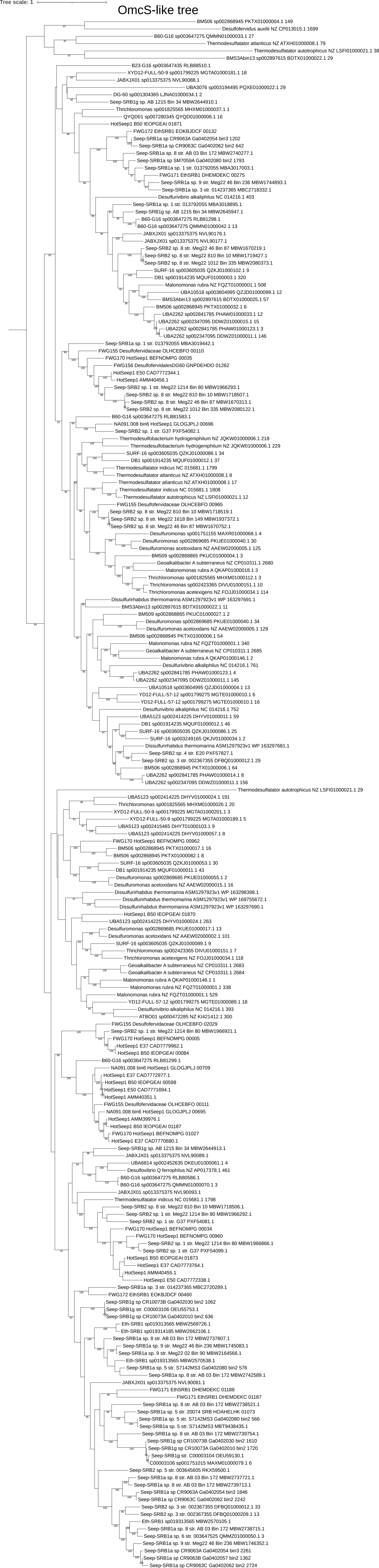

Supplement: S1 Data — Gene_trees_from_syntrophic_SRB.zip. (ZIP) [file pbio.3002292.s035.zip › S1_Data_Gene_trees_from_syntrophic_SRB/OmcSlike_tree.pdf]

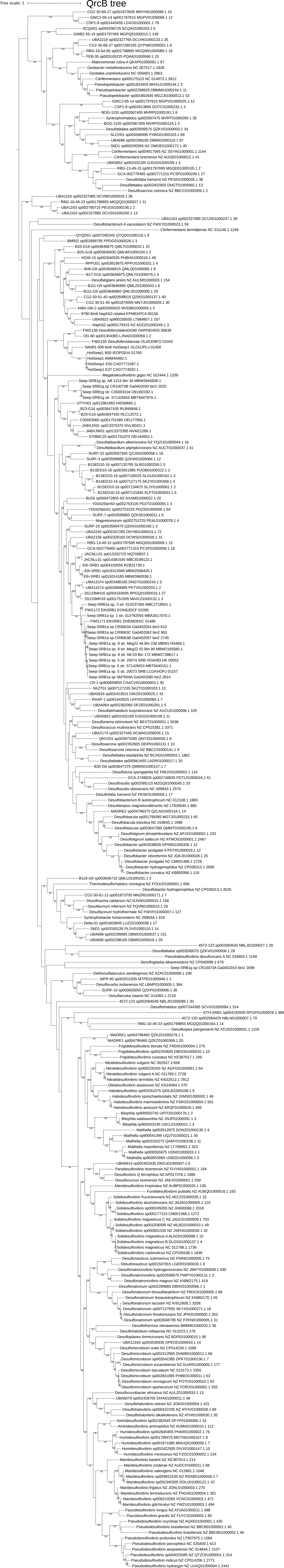

Supplement: S1 Data — Gene_trees_from_syntrophic_SRB.zip. (ZIP) [file pbio.3002292.s035.zip › S1_Data_Gene_trees_from_syntrophic_SRB/QrcB_tree.pdf]

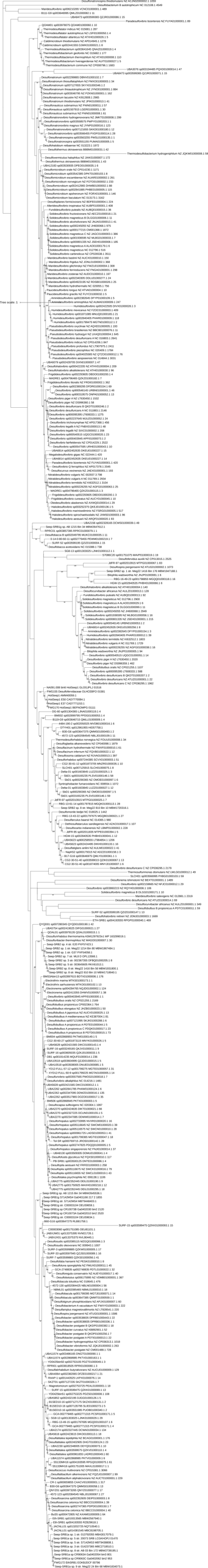

Supplement: S1 Data — Gene_trees_from_syntrophic_SRB.zip. (ZIP) [file pbio.3002292.s035.zip › S1_Data_Gene_trees_from_syntrophic_SRB/AprA_tree.pdf]

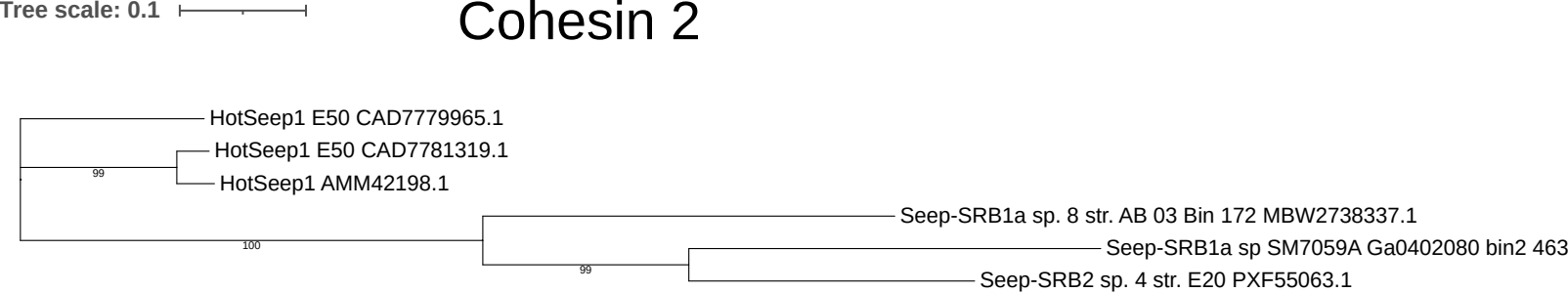

Supplement: S1 Data — Gene_trees_from_syntrophic_SRB.zip. (ZIP) [file pbio.3002292.s035.zip › S1_Data_Gene_trees_from_syntrophic_SRB/adhesin14_cohesin2_iqtree.pdf]
