# Supplementary material for: Physiological potential and evolutionary trajectories of syntrophic sulfate-reducing bacterial partners of anaerobic methanotrophic archaea
Source: PLoS Biol. 2023 Sep 25;21(9):e3002292. doi: 10.1371/journal.pbio.3002292 (PMC10553843; doi:10.1371/journal.pbio.3002292)
Supplement: S1 Text — (S1_text_Proposal_for_formal_nomenclature.pdf). (PDF) [file pbio.3002292.s034.pdf]

Supplementary Information

Section	Page
Proposed formal nomenclature for Seep-SRB1a, Seep-SRB1g and Seep-SRB2	1

Proposed formal nomenclature for Seep-SRB1a, Seep-SRB1g and Seep-SRB2

For each of the these clades, type material has been assigned with a high quality metagenome assembled genome. As far as possible, the standards for type material as identified in Chuvochina *et al.* were followed and the metrics for genome quality are detailed in the table below.

Clade	Species	Assembly	Genome size(bp)	Completeness	Contamination	Strain Heterogeneity	Presence of 23S, 16S rRNA	Presence of tRNA	Number of contigs	Quality Score[1]	Geographic location	Reference
Seep-SRB2	Desulfomithrium nov sp. 2	JAJSZR000000000	2622393	93.73	0.68	75	16S, 23S	18	220	91.01	Vent sediment Pescadero Basin, Gulf of California	This study
Seep-SRB1g	Desulfomellonium nov sp. 1	JAJSZX000000000	3489866	99.17	0.65	0	16S, 23S	18	291	96.57	cold seep, Costa Rica Margin	This study
Seep-SRB1a	Syntrophophila nov sp. 2	JAJSZT000000000	3859851	99.35	2.15	0	16S, 23S	19	170	90.75	cold seep, Costa Rica Margin	This study
Seep-SRB1a	Syntrophophila nov sp. 4	JAJSZP000000000	2570901	92.1	4.73	25	no	19	251	73.18	cold seep, Santa Monica Basin, California, USA	This study
Seep-SRB1a	Syntrophophila nov sp. 5	JAJSZW000000000	3861344	89.65	1.29	0	no	15	257	84.49	cold seep, Santa Monica Basin, California, USA	This study

[1]Quality score = Completeness – 4*contamination

1. Proposed formal name for organisms from the clade Seep-SRB2

Seep-SRB2 is at least a genus level clade within the order Dissulfuribacteriales, forming a family with the genus DTY0O1. We propose the formal name Desulfomithrium for Seep-SRB2 that will propagate to the family level clade Desulfomithraceae.

Description of the genus *Desulfomithrium* gen. nov.

Desulfomithrium (De.sul.fo.mith.rium., L. pref. *de*, off; L. neut. n. *sulfur*, sulfur; Sanskrit neut. n. mithram (मित्रम्), friend; N.L. neut. n. *Desulfomithrium* a sulfate reducing friend or syntrophic partner). All species within this genus who have been identified using 16S rRNA sequence similarity, and microscopy appear to partner anaerobic methanotrophic archaea (ANME) and form multi-cellular consortia with ANME. Their genomes indicate the presence of a sulfate reducing pathway, the Wood-Ljundahl pathway carbon fixation pathway, and a multi-heme cytochrome cluster that is hypothesized to accept electrons from ANME directly. We designate the type species for this genus as *Candidatus* Desulfomithrium sp. nov. 2.

Description of the species *Desulfomithrium* sp. nov. 2

Desulfomithrium (De.sul.fo.mith.rium., L. pref. *de*, off; L. neut. n. *sulfur*, sulfur; Sanskrit neut. n. mithram (मित्रम्), friend; N.L. neut. n. *Desulfomithrium* a sulfate reducing friend or syntrophic partner). This species was identified using 16S rRNA sequence similarity, and an average nucleotide identity (ANI) cut-off of 95 % to distinguish it from other species of the genus

D*esulfomithrium*. This species was identified in hydrothermal vent sediment from South Pescadero Basin. We designate the type material for this species as the genome, JAJSZR000000000.

2. Proposed formal name for organisms from the clade Seep-SRB1a

Seep-SRB1a is a genus level clade within a family that includes the species Eth-SRB1. The proposed formal name will propogate to the family level clade Syntrophophilaceae.

Description of the genus *Syntrophophila* gen. nov.

Syntrophophila (Syn.tro.pho.phi.la, Gr. pref. *syn*, with; Gr. pref., *tropho*, nourish; Gr. female. n. *phila*, loving, N.L. fem. n. *Syntrophophila* bacteria that loves syntrophic relationships with anaerobic methanotrophic archaea. All species within this genus who have been identified using 16S rRNA sequence similarity or whole genome phylogeny, and microscopy appear to partner anaerobic methanotrophic archaea (ANME) and form multi-cellular consortia with ANME. Their genomes indicate the presence of a sulfate reducing pathway, the Wood-Ljundahl pathway carbon fixation pathway, and a multi-heme cytochrome cluster that is hypothesized to accept electrons from ANME directly. We designate the type species for this genus as *Candidatus* Syntrophophila sp. nov. 2.

Description of the species *Syntrophophila* sp. nov. 2

Syntrophophila (Syn.tro.pho.phi.la, Gr. pref. *syn*, with; Gr. pref., *tropho*, nourish; Gr. female. n. *phila*, loving, N.L. fem. n. *Syntrophophila* bacteria that loves syntrophic relationships with anaerobic methanotrophic archaea. This species' genome was sequenced from single aggregates of ANME and SRB sorted by FACS after labeling by BioOrthogonal Non-Canonical Amino acid Tagging (BONCAT). The original environmental sample came from methane seep sediment off the coast of Costa Rica. This species was shown to partner ANME-2c. Additionally, this species includes a cobalamin biosynthesis pathway. We designate the type material as the genome with the accession JAJSZT000000000.

Description of the species *Syntrophophila* sp. nov. 4

Syntrophophila (Syn.tro.pho.phi.la, Gr. pref. *syn*, with; Gr. pref., *tropho*, nourish; Gr. female. n. *phila*, loving, N.L. fem. n. *Syntrophophila* bacteria that loves syntrophic relationships with anaerobic methanotrophic archaea. This species was identified from a microbial mat in a methane seep in Santa Monica Basin, California. We designate the type material as the genome with the accession JAJSZP000000000.

Description of the species *Syntrophophila* sp. nov. 5

Syntrophophila (Syn.tro.pho.phi.la, Gr. pref. *syn*, with; Gr. pref., *tropho*, nourish; Gr. female. n. *phila*, loving, N.L. fem. n. *Syntrophophila* bacteria that loves syntrophic relationships with anaerobic methanotrophic archaea. This species' genome was sequenced from single aggregates of ANME and SRB sorted by FACS after labeling by BioOrthogonal Non-Canonical Amino acid Tagging (BONCAT). The original environmental sample came from methane seep sediment off the coast of Costa Rica. This species was shown to partner ANME-2a. We designate the type material as the genome with the accession JAJSZW000000000.

3. Proposed formal name for organisms from the clade Seep-SRB1g

Seep-SRB1g is a species within an order level clade with Seep-SRB1c. This will propagate to the family level as Desulfomellonaceae and order level name as Desulfomellonales.

Description of the genus *Desulfomellonium* gen. nov.

Desulfomellonium (De.sul.fo.mel.lo.nium. L. pref. *de*, off; L. neut. n. *sulfur*, sulfur; from the language Sindarin created by J.R.R.Tolkien, n. *mellon,* friend; N.L. neut. n. *Desulfomellonium*, a sulfate reducing friend or syntrophic partner.) All species within this genus as identified by 16S rRNA sequence similarity or microscopy have been shown to partner ANME-2b. Their genomes indicate the presence of a sulfate reducing pathway, the Wood-Ljundahl pathway carbon fixation pathway, and a multi-heme cytochrome cluster that is hypothesized to accept electrons from ANME directly. We designate the type species for this genus as *Candidatus* Desulfomellonium sp. nov. 2.

Description of the species *Desulfomellonium* sp. nov. 1

Desulfomellonium (De.sul.fo.mel.lo.nium. L. pref. *de*, off; L. neut. n. *sulfur*, sulfur; from the language Sindarin created by J.R.R.Tolkien, n. *mellon,* friend; N.L. neut. n. *Desulfomellonium*, a sulfate reducing friend or syntrophic partner.) This species was identified by 16S rRNA sequence similarity. This species was identified by sequencing a single aggregate of ANME and SRB sorted by FACS after labelling by BONCAT. The original environmental sample came from a methane seep sediment off the coast of Costa Rica. We designate the type material as the genome with the accession JAJSZX000000000.
