# Supplementary material for: Physiological potential and evolutionary trajectories of syntrophic sulfate-reducing bacterial partners of anaerobic methanotrophic archaea
Source: PLoS Biol. 2023 Sep 25;21(9):e3002292. doi: 10.1371/journal.pbio.3002292 (PMC10553843; doi:10.1371/journal.pbio.3002292)

AprB

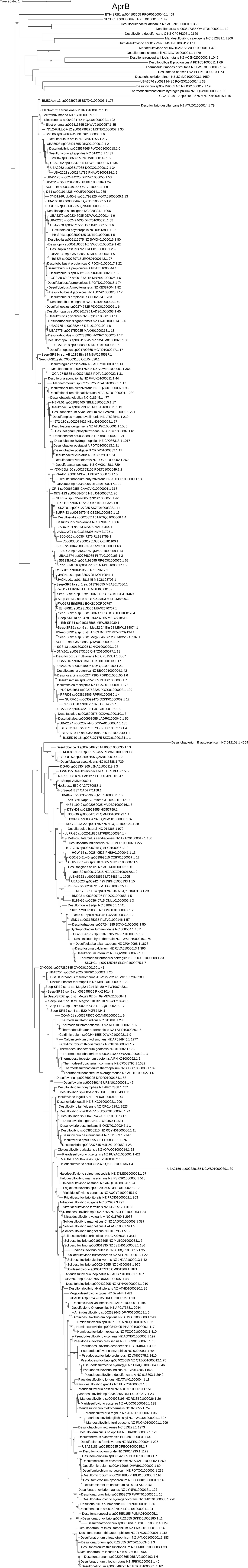

Supplement: S1 Data — Gene_trees_from_syntrophic_SRB.zip. (ZIP) [file pbio.3002292.s035.zip › S1_Data_Gene_trees_from_syntrophic_SRB/AprB_tree.pdf]
