# Supplementary material for: Physiological potential and evolutionary trajectories of syntrophic sulfate-reducing bacterial partners of anaerobic methanotrophic archaea
Source: PLoS Biol. 2023 Sep 25;21(9):e3002292. doi: 10.1371/journal.pbio.3002292 (PMC10553843; doi:10.1371/journal.pbio.3002292)
Supplement: S1 Data — Gene_trees_from_syntrophic_SRB.zip. (ZIP) [file pbio.3002292.s035.zip › S1_Data_Gene_trees_from_syntrophic_SRB/DsrA_tree_with_curated_database.pdf]

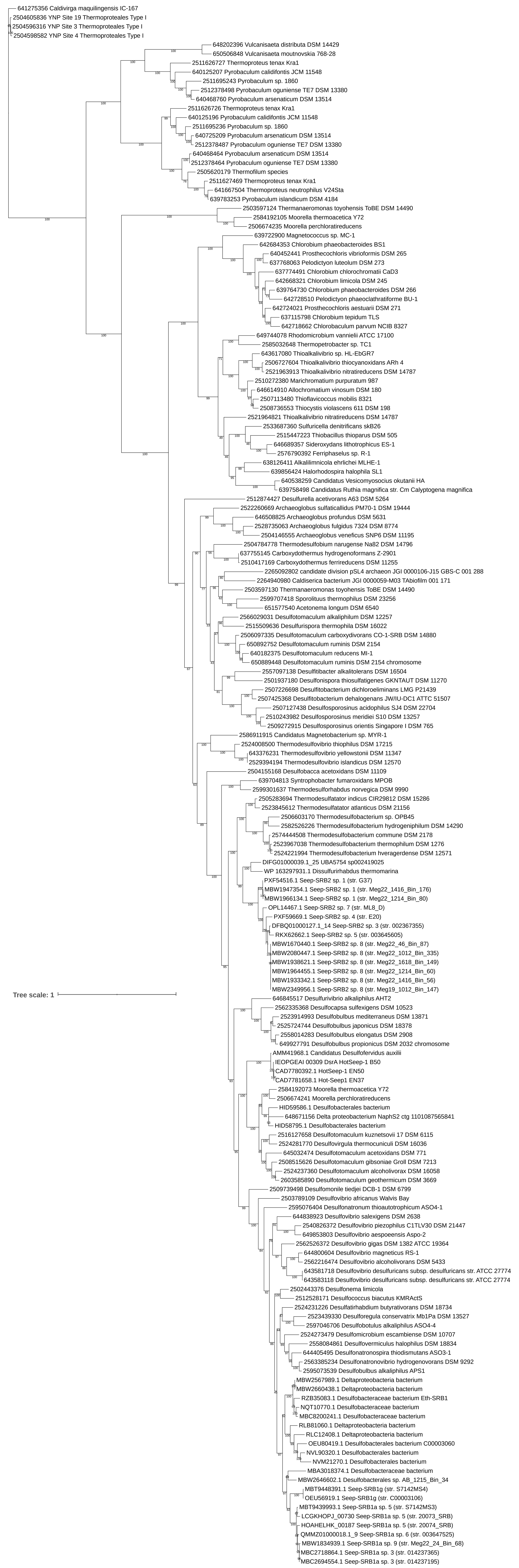
641275356 Caldivirga maquilingensis IC-167
2504605836 YNP Site 19 Thermoproteales Type I
2504596316 YNP Site 3 Thermoproteales Type I
2504598582 YNP Site 4 Thermoproteales Type I
648202396 Vulcanisaeta distributa DSM 14429
650506848 Vulcanisaeta moutnovskia 768-28
2511626727 Thermoproteus tenax Kra1
640125207 Pyrobaculum calidifontis JCM 11548
2511695243 Pyrobaculum sp. 1860
2512378498 Pyrobaculum oguniense TE7 DSM 13380
640468760 Pyrobaculum arsenaticum DSM 13514
2511626726 Thermoproteus tenax Kra1
640125196 Pyrobaculum calidifontis JCM 11548
2511695236 Pyrobaculum sp. 1860
640725209 Pyrobaculum arsenaticum DSM 13514
2512378487 Pyrobaculum oguniense TE7 DSM 13380
640468464 Pyrobaculum arsenaticum DSM 13514
2512378464 Pyrobaculum oguniense TE7 DSM 13380
2505620179 Thermofilum species
2511627469 Thermoproteus tenax Kra1
641667504 Thermoproteus neutrophilus V24Sta
639783253 Pyrobaculum islandicum DSM 4184
2503597124 Thermanaeromonas toyohensis ToBE DSM 14490
2584192105 Moorella thermoacetica Y72
2506674235 Moorella perchloratireducens
639722900 Magnetococcus sp. MC-1
642684353 Chlorobium phaeobacteroides BS1
640452441 Prosthecochloris vibrioformis DSM 265
637768063 Pelodictyon luteolum DSM 273
637774491 Chlorobium chlorochromatii CaD3
642668321 Chlorobium limicola DSM 245
639764730 Chlorobium phaeobacteroides DSM 266
642728510 Pelodictyon phaeoclathratiforme BU-1
642724021 Prosthecochloris aestuarii DSM 271
637115798 Chlorobium tepidum TLS
642718662 Chlorobaculum parvum NCIB 8327
649744078 Rhodomicrobium vannielii ATCC 17100
2585032648 Thermopetrobacter sp. TC1
643617080 Thioalkalivibrio sp. HL-EbGR7
2506727604 Thioalkalivibrio thiocyanoxidans ARh 4
2521963913 Thioalkalivibrio nitratireducens DSM 14787
2510272380 Marichromatium purpuratum 987
646614910 Allochromatium vinosum DSM 180
2507113480 Thioflavicoccus mobilis 8321
2508736553 Thiocystis violascens 611 DSM 198
2521964821 Thioalkalivibrio nitratireducens DSM 14787
2533687360 Sulfuricella denitrificans skB26
2515447223 Thiobacillus thioparus DSM 505
646689357 Sideroxydans lithotrophicus ES-1
2576790392 Ferriphaselus sp. R-1
638126411 Alkalilimnicola ehrlichei MLHE-1
639856424 Halorhodospira halophila SL1
640538259 Candidatus Vesicomyosocius okutanii HA
639758498 Candidatus Ruthia magnifica str. Cm Calyptogena magnifica
2512874427 Desulfurella acetivorans A63 DSM 5264
2522260669 Archaeoglobus sulfaticallidus PM70-1 DSM 19444
646508825 Archaeoglobus profundus DSM 5631
2528735063 Archaeoglobus fulgidus 7324 DSM 8774
2504146555 Archaeoglobus veneficus SNP6 DSM 11195
2504784778 Thermodesulfobium narugense Na82 DSM 14796
637755145 Carboxydothermus hydrogenoformans Z-2901
2510417169 Carboxydothermus ferrireducens DSM 11255
2265092802 candidate division pSL4 archaeon JGI 0000106-J15 GBS-C 001 288
2264940980 Caldiserica bacterium JGI 0000059-M03 TAbiofilm 001 171
2503597130 Thermanaeromonas toyohensis ToBE DSM 14490
2599707418 Sporolituus thermophilus DSM 23256
651577540 Acetonema longum DSM 6540
2566029031 Desulfotomaculum alkaliphilum DSM 12257
2515509636 Desulfurispora thermophila DSM 16022
2506097335 Desulfotomaculum carboxydivorans CO-1-SRB DSM 14880
650892752 Desulfotomaculum ruminis DSM 2154
640182375 Desulfotomaculum reducens MI-1
650889448 Desulfotomaculum ruminis DSM 2154 chromosome
2557097138 Desulfitibacter alkalitolerans DSM 16504
2501937180 Desulfonispora thiosulfatigenes GKNTAUT DSM 11270
2507226698 Desulfitobacterium dichloroeliminans LMG P21439
2507425368 Desulfitobacterium dehalogenans JW/IU-DC1 ATTC 51507
2507127438 Desulfosporosinus acidophilus SJ4 DSM 22704
2510243982 Desulfosporosinus meridiei S10 DSM 13257
2509272915 Desulfosporosinus orientis Singapore I DSM 765
2586911915 Candidatus Magnetobacterium sp. MYR-1
2524008500 Thermodesulfovibrio thiophilus DSM 17215
643376231 Thermodesulfovibrio yellowstonii DSM 11347
2529394194 Thermodesulfovibrio islandicus DSM 12570
2504155168 Desulfobacca acetoxidans DSM 11109
639704813 Syntrophobacter fumaroxidans MPOB
2599301637 Thermodesulforhabdus norvegica DSM 9990
2505283694 Thermodesulfatator indicus CIR29812 DSM 15286
2523845612 Thermodesulfatator atlanticus DSM 21156
2506603170 Thermodesulfobacterium sp. OPB45
2582526226 Thermodesulfobacterium hydrogeniphilum DSM 14290
2574444508 Thermodesulfobacterium commune DSM 2178
2523967038 Thermodesulfobacterium thermophilum DSM 1276
2524221994 Thermodesulfobacterium hveragerdense DSM 12571
DIFG01000039.1_25 UBA5754 sp002419025
WP 163297931.1 Dissulfurirhabdus thermomarina
PXF54516.1 Seep-SRB2 sp. 1 (str. G37)
MBW1947354.1 Seep-SRB2 sp. 1 (str. Meg22_1416_Bin_176)
MBW1966134.1 Seep-SRB2 sp. 1 (str. Meg22_1214_Bin_80)
OPL14467.1 Seep-SRB2 sp. 7 (str. ML8_D)
PXF59669.1 Seep-SRB2 sp. 4 (str. E20)
DFBQ01000127.1_14 Seep-SRB2 sp. 3 (str. 002367355)
RKX62662.1 Seep-SRB2 sp. 5 (str. 003645605)
MBW1670440.1 Seep-SRB2 sp. 8 (str. Meg22_46_Bin_87)
MBW2080447.1 Seep-SRB2 sp. 8 (str. Meg22_1012_Bin_335)
MBW1938621.1 Seep-SRB2 sp. 8 (str. Meg22_1618_Bin_149)
MBW1964455.1 Seep-SRB2 sp. 8 (str. Meg22_1214_Bin_60)
MBW1933342.1 Seep-SRB2 sp. 8 (str. Meg22_1416_Bin_56)
MBW2349956.1 Seep-SRB2 sp. 8 (str. Meg19_1012_Bin_147)
Tree scale: 1
646845517 Desulfurivibrio alkaliphilus AHT2
2562335368 Desulfocapsa sulfexigens DSM 10523
2523914993 Desulfobulbus mediterraneus DSM 13871
2525724744 Desulfobulbus japonicus DSM 18378
2558014283 Desulfobulbus elongatus DSM 2908
649927791 Desulfobulbus propionicus DSM 2032 chromosome
AMM41968.1 Candidatus Desulfofervidus auxilii
IEOPGEAI 00309 DsrA HotSeep-1 B50
CAD7780392.1 HotSeep-1 EN50
CAD7781658.1 Hot-Seep1 EN37
2584192073 Moorella thermoacetica Y72
2506674241 Moorella perchloratireducens
HID59586.1 Desulfobacterales bacterium
648671156 Delta proteobacterium NaphS2 ctg 1101087565841
HID58795.1 Desulfobacterales bacterium
2516127658 Desulfotomaculum kuznetsovii 17 DSM 6115
2524281770 Desulfovirgula thermocuniculi DSM 16036
645032474 Desulfotomaculum acetoxidans DSM 771
2508515626 Desulfotomaculum gibsoniae Groll DSM 7213
2524237360 Desulfotomaculum alcoholivorax DSM 16058
2603585890 Desulfotomaculum geothermicum DSM 3669
2509739498 Desulfomonile tiedjei DCB-1 DSM 6799
2503789109 Desulfovibrio africanus Walvis Bay
2595076404 Desulfonatronum thioautotrophicum ASO4-1
644838923 Desulfovibrio salexigens DSM 2638
2540826372 Desulfovibrio piezophilus C1TLV30 DSM 21447
649853803 Desulfovibrio aespoeensis Aspo-2
2562526372 Desulfovibrio gigas DSM 1382 ATCC 19364
644800604 Desulfovibrio magneticus RS-1
2562216474 Desulfovibrio alcoholivorans DSM 5433
643581718 Desulfovibrio desulfuricans subsp. desulfuricans str. ATCC 27774
643583118 Desulfovibrio desulfuricans subsp. desulfuricans str. ATCC 27774
2502443376 Desulfonema limicola
2512528171 Desulfococcus biacutus KMRActS
2524231226 Desulfatirhabdium butyrativorans DSM 18734
2523439330 Desulforegula conservatrix Mb1Pa DSM 13527
2597046706 Desulfobotulus alkaliphilus ASO4-4
2524273479 Desulfomicrobium escambiense DSM 10707
2558084861 Desulfovermiculus halophilus DSM 18834
644405495 Desulfonatronospira thiodismutans ASO3-1
2563385234 Desulfonatronovibrio hydrogenovorans DSM 9292
2595073539 Desulfobulbus alkaliphilus APS1
MBW2567989.1 Deltaproteobacteria bacterium
MBW2660438.1 Deltaproteobacteria bacterium
RZB35083.1 Desulfobacteraceae bacterium Eth-SRB1
NQT10770.1 Desulfobacteraceae bacterium
MBC8200241.1 Desulfobacteraceae bacterium
RLB81060.1 Deltaproteobacteria bacterium
RLC12408.1 Deltaproteobacteria bacterium
OEU80419.1 Desulfobacterales bacterium C00003060
NVL90320.1 Desulfobacterales bacterium
NVM21270.1 Desulfobacterales bacterium
MBA3018374.1 Desulfobacteraceae bacterium
MBW2646602.1 Desulfobacterales sp. AB_1215_Bin_34
MBT9448391.1 Seep-SRB1g (str. S7142MS4)
OEU56919.1 Seep-SRB1g (str. C00003106)
MBT9439993.1 Seep-SRB1a sp. 5 (str. S7142MS3)
LCGKHOPJ_00730 Seep-SRB1a sp. 5 (str. 20073_SRB)
HOAHELHK_00187 Seep-SRB1a sp. 5 (str. 20074_SRB)
QMMZ01000018.1_9 Seep-SRB1a sp. 6 (str. 003647525)
MBW1834939.1 Seep-SRB1a sp. 9 (str. Meg22_24_Bin_68)
MBC2718864.1 Seep-SRB1a sp. 3 (str. 014237365)
MBC2694554.1 Seep-SRB1a sp. 3 (str. 014237195)
