# Supplementary material for: Physiological potential and evolutionary trajectories of syntrophic sulfate-reducing bacterial partners of anaerobic methanotrophic archaea
Source: PLoS Biol. 2023 Sep 25;21(9):e3002292. doi: 10.1371/journal.pbio.3002292 (PMC10553843; doi:10.1371/journal.pbio.3002292)
Supplement: Attachment — Submitted filename: Response_to_reviewer_comments_final.pdf [file pbio.3002292.s037.pdf]

Reviewer #1: This manuscript focuses on understanding the adaptations of syntrophic sulfate reducing bacteria (SRB) that make consortia with anaerobic methanotrophic archaea (ANME). They focus on four different clades of SRB that perform sulfate-coupled anaerobic oxidation of methane (AOM) - HotSeep-1, Seep-SRB2, Seep-SRB1a and Seep-SRB1g - and perform a genomic comparison with their nearest evolutionary neighbours. This is a very logical approach that indeed reveals several differences that are pertinent to the syntrophic associations and seem to be unique to each partnership and to have evolved independently. This is an interesting study that provides very relevant information, such as the recruitment of multiheme cytochrome proteins for DIET, the involvement of specific membrane complexes in the respiratory chain, some nutritional dependencies and the involvement of the extracellular matrix in the interaction.

I found the work to be interesting and carefully performed, and have only a few questions that I think merit further discussion:

We thank the reviewer for their careful reading, insightful questions and positive assessment of the manuscript.

- Why is it proposed that the Tmc complex reduces DsrC in the HotSeep-1, Seep-SRB1a and Seep- SRB1g, when all these organisms contain the DsrMKJOP complex, which is considered to be the physiological electron donor of DsrC. Why is a special focus put on Tmc? This is not clear throughout the manuscript.

Thanks for pointing this out. We suspect that both Tmc and Dsr are donors to DsrC. We note that DsrMKJOP also can donate electrons to DsrC, ”the DsrMKJOP complexes transfers electrons from quinones to DsrC and through DsrC to DsrAB.” (lines 379-380). In previous experiments, Tmc and Dsr are both highly expressed (Yu et al. ISME 2020). The special emphasis on Tmc is because of the divergence of Tmc from canonical SRB. We have added a line to make this more clear. “Curiously, the Tmc complex in Seep-SRB1a and Seep-SRB1g are divergent from Tmc in non-syntrophic SRB (**Supplementary Figure 8**) and TmcA is absent in the operons encoding for Tmc. This absence suggests that Tmc has been adapted to use a different electron donor than in *Desulfovibrio vulgaris*, and is consistent with the fact that the electron donor for Seep-SRB1a and Seep-SRB1g is not hydrogen or formate but, electrons from anaerobic methanotrophic archaea.“ (Lines 371-375)

- Also it is stated that Tmc is heavily adapted for DIET. Why??

TmcA is missing from the operon encoding for TmcBCD in Seep-SRB1g and Seep-SRB1a. This is rarely the case for TmcABCD in other SRB. Since TmcA acts as the site of electron input into the Tmc complex, this absence suggests that Tmc has been adapted to use a different electron donor than *$TpIc_3$*, the electron donor to Tmc in *Desulfovibrio vulgaris*. Also, refer above.

- The TmcD of Seep-SRB1a and Seep-SRB1g appear to differ from that of other SRB (Figure S8). What is the difference?

We are really interested in this question as well! In *Desulfovibrio*, TmcD is predicted to act as a peripheral membrane protein based on its tryptophan-rich domain (Pereira *et al.* 2006). TmcD in Seep-SRB1a and Seep-SRB1g also retain the conserved tryptophan residues so we suspect that Seep-SRB1a/g TmcD can still play this role. We tried to

investigate the other changes that were found in this protein, with a multiple sequence alignment (made available in online supplementary data) and structural modeling. A TmcD structural model from Seep-SRB1a is made available as Supplementary Figure 19. There are several conserved residues that differentiate this clade from other SRB such as a few conserved cysteines (highlighted in green in Supplementary Figure 19). However, it is not clear what role these divergent residues, specifically these cysteines play. They are not structurally close enough to form disulfide bonds or bind other co-factors. Without more biochemical information, it is hard to know what the conserved residues in this protein are doing.

- Also it is stated that Qrc is the major site of energy conservation in this respiratory pathway. What is the basis for this statement? I cannot tell why it should be the major site. Qmo and Dsr will also likely contribute, and possibly others.

With the use of quinones and quinols as an electron donor, there are 2 ways to conserve energy across the inner membrane either by movement of protons or electrons across the membrane in a single protein complex, or by a loop formed with two complexes, one in which protons can be taken up at the cytoplasm for one complex and released at the periplasm for the second complex. Qrc appears to form an example of the first type, and is electrogenic because it appears to moves charges across the membrane (Duarte *et al.* Nature Communications 2018) with a putative proton channel from the cytoplasmic side to a periplasmic quinone binding site, so that electrons from a periplasmic electron carrier can be used to reduce a quinone at the periplasmic binding site. It will allow for the conservation of energy regardless of whether quinols are oxidized at the periplasmic or cytoplasmic side for other quinol oxidizing complexes such as Qmo or Dsr. This is why we refer to it as the major site of energy conservation. But, you are correct that Qmo and Dsr could contribute to energy conservation through a redox loop, since we do not yet know where the quinol binding site for these complexes are. We have therefore amended this statement to say “Qrc is known to be important for energy conservation in this respiratory pathway (Line 375)” rather than to say that it is the major site of energy conservation.

- Please describe better what is the QmoC-fusion protein that also binds heme c. Is it also predicted to bind hemes b and FeS clusters like QmoC? And how many hemes c? From the description it does not seem to have proteins that would interact with DsrC, so the proposal that it transfers electrons from cytochromes c and DsrC does not seem justitified.

The QmoC-fusion protein contains three hemes *c* and a CCG domain, also found in proteins such as in TmcB and HdrB. HdrB was recently shown to interact with DsrC (Ferreira et al. 2023). We think that periplasmic cytochromes *c* could interact with the domain containing 3 hemes *c* and DsrC could interact with the CCG containing domain. We amended the description of the complex to the following, ”HotSeep-1 genomes contain a complex that involves an HdrA subunit and a QmoC-fusion protein that also binds hemes c and contains a CCG domain similar to that found in HdrB and TmcB predicted to interact with DsrC. This complex would likely transfer electrons from cytochromes c to the DsrC (AMM42179.1-AMM42180.1) or perhaps to a ferredoxin.” (Lines 399-400)

- Lines 437 and 445: Why would electrons go from DsrC to NAD(P)H or ferredoxin? This does not make sense. Rnf or Mrp are not expected to interact with DsrC.

This is poorly phrased perhaps. We meant that the route of electron transfer from periplasmic cytochromes c through the identified inner membrane complexes leads to the reduction of either membrane-bound quinols or to the cytoplasmic electron carrier DsrC. For e.g., Tmc reduces DsrC, and Qrc reduces quinols and Qmo and DsrMKJOP reduces AprAB and DsrC respectively. None of these complexes interact with NADPH or ferredoxin. Yet, NADPH and ferredoxin must be the electron donors to carbon and nitrogen fixation pathways, as biochemical characterization of those pathways has shown in the past. Therefore, there must be complexes that transfer electrons from DsrC to NAD(P)H or ferredoxin. One example of such a complex is Flx-Hdr for e.g., which transfers electrons from DsrC to NADH (Ramos et al. 2015). And if reduced DsrC can be oxidized by Flx-Hdr to generate NADH, then the Rnf complex could be used to generate ferredoxin from NADH. We have explicitly identified Flx-Hdr as an example in the text and hope that it makes it more clear. "The transfer of electrons from DsrC to these reductants likely happens through the action of protein complexes like Flx-Hdr that interact with DsrC and NADH. NADH and ferredoxin can then interconverted with the dissipation or generation of sodium motive force using membrane-bound Rnf and Mrp in Seep-SRB1a, Seep-SRB1g and Hot-Seep1." (Lines 421-425)

- A large abundance of HdrA-like proteins is also seen in non-syntrophic sulfate reducers and many other anaerobes. It is not clear if this is specific or relevant for the syntrophic ones.

We agree that the use of HdrA-like proteins is not unique to syntrophic SRB, and is found in many anaerobes and non-syntrophic SRB. We have however noted that at least in the case of Seep-SRB1g, the metF-HdrABC is not found in the nearest evolutionary neighbor Seep-SRB1c. But we have also acknowledged in the text that the presence of a number of putative bifurcating complexes may be an adaptation to low-energy environments and not unique to syntrophy. "While the capability of electron bifurcation by these enzyme complexes needs to be biochemically confirmed, the possibility of a high number of bifurcating complexes, especially those connected to the carbon fixation pathway, in the genomes of syntrophic SRB partners of ANME is compelling. It could be argued that this is a natural adaptation to growth in very low energy environments or to low-energy metabolism. In fact, some of these complexes are present in other bacteria of the order Desulfofervidales and genus Eth-SRB1. These adaptations could provide an additional energetic benefit for the syntrophic lifestyle, itself an adaptation to low-energy environments." (Lines 462-448)

Reviewer #2: When I got the invitation to review this manuscript titled "Physiological adaptation of sulfate reducing bacteria in syntrophic partnership with anaerobic methanotrophic archaea", I was excited to read about new discoveries on the physiology of this interesting syntrophic partnership from one of the world leading lab on the physiology of ANME/SRB consortia. However, after reading the paper I was surprised since the paper does not report any physiological experiments or data but is

rather a predictive genomic analysis of existing (And some new) sequence data. But, predicting physiology from genomes is not the same thing as actual physiological studies. Thus, I found the way the manuscript is presented it is a bit misleading in this sense. The paper is very long, and reads for the most part like a review article rather than an original research article (for example, the manuscript has 115 citations).

For most of the manuscript, the focus tends to be heavily on results of other studies that did physiology. Even expression analysis has already been done (refs 22, 24) and the manuscript seems to rely heavily on those earlier studies to make the points. This gives more the impression of a review article. These prior physiology studies are discussed at length and it seems like the manuscript tries to use those prior physiology studies to try and patch together a new story on physiology, using their genomic data. There has been a lot of papers on this general topic, and actually the same group published a paper last year in PLoS Biology that also used FACS sorting and genomes of ANME/SRB consortia. So, for me the impact of this new manuscript is a bit dliuted.

https://journals.plos.org/plosbiology/article?id=10.1371/journal.pbio.3001508

Here are just a few examples of how the papers title and conclusions are a bit misleading since there is no physiology reported in the paper (only predictions from genomes/metagenomes) :

The title starts with: "Physiological adaptation of sulfate reducing bacteria..."

line 139: "In this work, we described a physiological framework ..... "

line 146: "Our study explored the diverse physiological strategies that underlie..."

line 668 : "Physiological adaptation of syntrophic SRB to partnerships with ANME"

I think the manuscript has potential to become interesting, for example if the authors re focused it more onto the aspect of convergent evolution of the SRB into the syntrophy with the ANME. And, the varying steps of dependence during the development of the syntrophy like the loss of hydrogenase in the SRB as they become more dependent on electrons from the methane from the ANME (this would maybe be an interesting idea to for the authors to follow up on). The HGT story and the cobalamin evolution was also a potentially interesting pursuit. However, as interesting as those things are they are buried in a long manuscript that is full of speculations on physiology from genome data. All of these interesting evolutionary aspects are comparative genomics (not physiology) and in my opinion the manuscript would benefit from a major re-structuring and re-messaging towards the evolutionary aspects (and away from physiology predictions) and resubmitted to a more evolutionary focused microbiology specific journal.

We thank the reviewer for the critical comments. Indeed, we wholeheartedly agree with the reviewer that more detailed experimental characterization on the physiological side of

these environmental slow growing consortia is needed for the field of AOM, which our lab and many others have worked towards in the past two decades. We appreciate the reviewer pointing out that the manuscript title ‘Physiological adaptation of sulfate reducing bacteria…’ may have set different expectations for its content. It was certainly not our intention to mislead. We have changed the title to ‘Identification of physiological potential and evolutionary traits of diverse syntrophic sulfate reducing bacterial partners of ANME using comparative genomics’, and hope this provides a more accurate reflection of this manuscript.

We believe this synthesis work, based on detailed genomic analysis of all known major representative syntrophic sulfate-reducing bacterial (SRB) partners of ANME archaea (the majority uncultured), is highly valuable in advancing our understanding of the key steps in SR-AOM syntrophy and specificity of interactions between ANME and SRB. This manuscript offers a critical framework and potential hypotheses to test in future studies using targeted experiments, much in the same way our recent 2022 PLOS Biology manuscript did by focusing on the phylogenomic characterization of the major methane oxidizing ANME archaea (Chadwick et al PLOS Biology). The enormous diversity of ANME-SRB partnerships observed in nature over the past ~25 years since their discovery (Hinrichs et al 1999, Boetius et al 2000, Orphan et al 2001)– are still poorly described in terms of their basic taxonomy, genome content, and ecophysiology. This necessitates a well vetted genomic and taxonomic framework for future progress in the field. In this spirit, the currently published Chadwick et al PLOS Biology and Murali et al. are intended to be companion articles covering both sides of the long enigmatic AOM syntrophy.

Below, we briefly summarize the impact of our paper focusing on the syntrophic SRB to the field in three aspects:

1. The bacterial partner clades involved in anaerobic methane oxidation have been traditionally poorly annotated. Papers surveying AOM in different ecosystems still cite the polyphyletic and taxonomically unrelated clade Seep-SRB1 as a potential partner that includes many bacteria that are not syntrophic ANME partners. This has been a long-standing problem in the field and will continue to hold the field back unless properly addressed. Our paper clearly establishes which of these clades are currently established partners (only Seep-SRB1a and Seep-SRB1g) and which are not. We then further support this by providing new genomic resources and detailed taxonomic profiling. (Figure1, Supplementary Figure 2)

2. We presented features of convergent and divergent evolution within such a partnership, which are mostly unrealized in the field. For example, we observed cobalamin auxotrophy within the ANME-2a/Seep-SRB1a partnerships but notably not the Seep-SRB1a which partner with a different family of ANME methanotroph, the ANME-2c. By detailed comparisons between partnered and non-partnered SRBs with varying taxonomic distances, we not only generate a strong knowledge base for future studies of AOM but also provide conceptual and methodological frameworks for future studies.

3. The current data suggests that ANME-SRB energy production relies on extracellular electron transfer (EET), but EET in sulfate reducing bacteria has not yet been a commonly observed phenomenon and is thus poorly understood. Our paper establishes a framework for understanding EET process within the SRB partners, which are critical for future discoveries and experimental characterizations.

In summary, we think that establishing a taxonomic, ecological/partnership framework is essential to facilitate future, in-depth investigation and understanding of the rich biological diversity within this partnership. For such a purpose, extensive biochemical explanations are necessary as is providing supporting context from existing transcriptomic and proteomic datasets. This expands the length of the paper but it is within the guidelines for publication in PLOS Biology. We have rewritten the abstract and a portion of the introduction (lines 74-121) to better emphasize the evolutionary aspect of this paper, and called attention to evolutionary relationships in the section 'Adaptation of syntrophic SRB to partnerships with ANME' (lines 694-711) but, because the purpose of this manuscript was not intended to be solely an evolutionary paper, we did not fully restructure the paper. We hoped to describe the ecology and evolutionary question in the introduction, provide a detailed discussion of the mechanisms involved in the syntrophic interaction in the results and discussion, followed by a summary of the phylogenetic analysis to discuss the evolutionary adaptation. Regarding the number of citations, we believe it does not necessarily affect the format of the paper, but we can reduce citations if deemed necessary by the editors.

Reviewer #3: This is a very long and quite well written paper describing genes, proteins and pathways present in syntrophic SRB that are involved in methane oxidation. The paper reads like a combination of a research paper and a review, drawing from lots of previous work to back up interpretations. I really enjoyed reading it and find the summaries, diagrams and interpretations helpful. It is often hard for me to tell how solid the data is supporting interpretations, but the authors tell a nice story and I am willing to go with it.
We thank the reviewer for the thorough reading of the manuscript, valuable comments and encouraging assessment of the manuscript.

Not a lot of comments considering how much information is here.

Abstract is not very illuminating. What results were found that show mechanisms of syntrophy? This should be rewritten to include some more interesting results. Rather than spending most of the abstract on what was done.
Thank you for drawing this to our attention. We have re-written the abstract to better highlight the key results as follows:
"Sulfate-coupled anaerobic oxidation of methane (AOM) is performed by multicellular consortia of anaerobic methanotrophic archaea (ANME) in obligate syntrophic partnership with sulfate-reducing bacteria (SRB). Diverse ANME and SRB clades co-associate but the physiological basis for their adaptation and diversification is not well

understood. In this work, we used comparative metagenomics and phylogenetics to investigate the metabolic adaptation among the four main syntrophic SRB clades (HotSeep-1, Seep-SRB2, Seep-SRB1a and Seep-SRB1g) and identified features associated with their syntrophic lifestyle that distinguish them from their non-syntrophic evolutionary neighbors in the phylum Desulfobacterota. We show that the protein complexes involved in direct interspecies electron transfer (DIET) from ANME to the SRB outer membrane are conserved between the syntrophic lineages. In contrast, the proteins involved in electron transfer within the SRB inner membrane differ between clades, indicative of convergent evolution in the adaptation to a syntrophic lifestyle. Our analysis suggests that in most cases this adaptation likely occurred after the acquisition of the DIET complexes in an ancestral clade and, involve horizontal gene transfers within pathways for electron transfer (CbcBA) and biofilm formation (Pel). We also provide evidence for unique adaptations within syntrophic SRB clades, which vary depending on the archaeal partner. Among the most widespread syntrophic SRB, Seep-SRB1a, subclades which specifically partner ANME-2a are missing the cobalamin synthesis pathway, suggestive of nutritional dependency on its partner, while closely related Seep-SRB1a partners of ANME-2c lack nutritional auxotrophies. Our work provides insight into the features associated with DIET-based syntrophy and the adaptation of SRB towards it."

52-53. Not sure that I would use the definition allowing for less energy investment for each partner. Second part of the sentence is not correct.
Upon review, we agree this should have been worded better. We intended to say that sharing resources in this way, can allow for less energy investment overall. For e.g. if one partner were to fix nitrogen, the other partner would expend less ATP than they would otherwise. But perhaps that can be interpreted that sharing resources benefits both partners simultaneously. We have rephrased this sentence to avoid that interpretation and hope that this is more clear. " Microorganisms benefit from sharing nutrients and electrons in this way, combining their resources and avoiding the need for both partners to expend energy for the synthesis of common nutrients[1,3]"

442-443. Reference is needed for this.
Thanks for noting that omission. A reference has now been added. In the revised manuscript, this is line 430.

517-526. This is really hard to follow. Should be shortened and smoothed out.
We have rephrased this section and added a reference to the appropriate figure. "Interestingly, the predicted cobalamin auxotrophy is not a uniform trait within the Seep SRB1a lineage, with cobamide biosynthesis genes present in the genomes of species Seep-SRB1a sp. 2 (n=3), Seep-SRB1a sp. 4 (n=1), and Seep-SRB1a sp. 9 (n=3) (Figure 5). Of the five species missing cobalamin biosynthesis pathways, two are verified ANME-2a partners. Of the four species containing cobalamin biosynthesis pathways, one is a verified ANME-2c partner and one was sequenced from a microbial mat that contains ANME-2c and ANME-2a (Figure 5). These patterns suggest that the

Seep-SRB1a partners of ANME-2a developed a nutritional auxotrophy that is specific to this partnership.” (Lines 503-509)

566-568. Not sure what the point of this sentence is. Should be reworded or removed.
The sentence has been removed

622-636. This should focus on proteins present in the syntrophs and absent in the non syntrophs. Clearly, there are many components of biofilms, but this would be more interesting if the specific ones used for syntrophy are described and then others mentioned.
Thank you for this note. This paragraph has been rephrased to emphasize the adhesins found in syntrophic SRB. (now lines 600-625)

650-666. this is good, but again should highlight mechanisms just present in syntrophs.
Thanks, we added a line to highlight the importance of secretion systems in syntrophy. “While secretion systems are not uncommon in non-syntrophic bacteria, the high degree of their conservation in ANME and the high levels of expression of secretion systems in the ANME-2/Seep-SRB1a[22] and ANME-2c/Seep-SRB2[24] partnerships suggest an important role for them in ANME-SRB syntrophy.” (Lines 638-641)

SFIG16. Colored lines need to be described.
We have added a description of the colored lines. “Lines in green and light teal are used to depict the partnerships between ANME-2c and verified species of Seep-SRB1a, and ANME-2a and verified species of Seep-SRB1a respectively Line in blue is used to depict the partnership between ANME-2b and Seep-SRB1g.”

681. Where are these trees? Authors should indicate where they are located here.
These are made available on the author’s github’s page. https://github.com/ranjani-m/syntrophic-SRB

696-697. I don't see how the latter point contributes to sequence divergence.
You are correct. There is no demonstrated correlation between horizontal gene transfer faster rates of gene evolution. The second half of this sentence has been removed.

700 Reword.
Reworded as follows. “With our analysis, we identified many genes and traits that are correlated with a syntrophic partnership with ANME, but it is less easy to identify whether they are essential. “ lines 678-679.

724-725. I do not understand this sentence regarding the Observed diversity.
We meant that if adaptation to partnerships with different ANME is not driving the species diversity within Seep-SRB1a and Seep-SRB2, something else must. We rephrased the lines preceding this line to say, “The greater diversity within the clades Seep-SRB1a and Seep-SRB2 may be a result of the larger number of partnerships with different ANME compared to a clade such as Seep-SRB1g. However, there is insufficient evidence to rule out the possibility of promiscuous partnership formation with

multiple ANME within each SRB species. In these cases, the observed species diversity must be driven by other factors." (Lines 704-708) We hope this makes it more clear.

731-733. this latter speculation is not necessary. Certainly possible, but no evidence. Not necessary but certainly interesting! In any case, the speculative sentence has been removed.
