# Supplementary material for: Physiological potential and evolutionary trajectories of syntrophic sulfate-reducing bacterial partners of anaerobic methanotrophic archaea
Source: PLoS Biol. 2023 Sep 25;21(9):e3002292. doi: 10.1371/journal.pbio.3002292 (PMC10553843; doi:10.1371/journal.pbio.3002292)
Supplement: Attachment — Submitted filename: Response_to_reviewers_07242023.docx [file pbio.3002292.s038.docx]

Reviewer #1: The authors have clarified some points relative to my questions, but I some of the arguments are not really convincing and some bias is being introduced in the interpretations. An important point regarding energy conservation is that the authors are too focused on small differences and disregarding the fact that the two essential complexes for sulfate reduction, QmoABC and DsrMKJOP, are conserved in the syntrophic SRB, and are certainly the important sites for energy conservation (along with Qrc). This should be made more clear rather than focusing mainly on Qrc.

We thank reviewer #1 for their careful reading of the manuscript and their detailed comments. We appreciate their clear expertise in the subject matter and are happy to make some changes as per their recommendation.

We made the following changes to emphasize that Qmo and Dsr are also important for pmf generation in addition to Qrc. **Lines 378-384 in revised manuscript.**

“Qrc is known to be important for energy conservation in this respiratory pathway. Protons are translocated by Qrc from the cytoplasmic side to the periplasmic active side. This movement of charges across the membrane leads to the generation of proton motive force (pmf) that can be utilized by ATP synthase to generate ATP[68]. In Seep-SRB1a, Seep-SRB1g and HotSeep-1, Qrc likely acts with the conserved DsrMKJOP and QmoABC to generate pmf. While purified Dsr and Qmo have not yet been shown to be electrogenic, it is expected that DsrMKJOP[69] and QmoABC[69,70] might be able to generate pmf by charge translocation.”

Role of the Tmc complex. The strong focus put on Tmc as the complex that reduces DsrC (lines 685-686) is in my view totally unfounded. It is based on the absence of TmcA, which happens only for SeepSRB-1g and SeepSRB-1a, whereas Hot-Seep1 has a full Tmc complex and no Tmc complex is present in SeepSRB2. It is possible that TmcA is encoded somewhere else in the genome in SeepSRB-1g and SeepSRB-1a, or is replaced by a different cytochrome c. On the other hand the argument that the absence of TmcA is a reflection of adaptation to syntrophic lifestyle or DIET does not make much sense because both Tmc and Qrc use the same electron donor, the TpIc3, which the authors propose is a amin palyer in periplasmic electron transfer also here, namely for Qrc. So, regardless of electrons coming from H2, formate or ANME they are likely transferred to TpIc3. So adaptation to syntropy does not need to imply loss of TmcA. So, I don't really see any evidence to support that Tmc will be more
relevant to energy conservation in the syntrophic than in free living SRB, specially when there is DsrMKJOP, the bona-fide electron donor to DsrC, universally present in SRB. This is misleading and should be corrected.

We do not mean to say that DsrMKJOP is less important than Tmc in syntrophic SRB to reduce DsrC. Indeed, both complexes are both expressed at equally high levels in HotSeep-1 (Krukenberg *et al.* Environmental Microbiology (2018)) and Seep-SRB1a (Yu *et al.* ISME 2021).

We are merely noting that the DsrMKJOP within syntrophic and non-syntrophic SRB are similar while the Tmc complexes within Seep-SRB1a and Seep-SRB1g are different from their nearest evolutionary neighbors that are free-living. The Seep-SRB1a and Seep-SRB1g operons are missing TmcA. While we noted the absence of TmcA, we also noted that the TmcD tree shows that the TmcD from Seep-SRB1a and Seep-SRB1g are different than those of their nearest evolutionary neighbors (Supplementary Figure 8). Therefore this is not an observation based only on the absence of TmcA. Please note that this is not true for the Tmc from HotSeep-1. HotSeep-1 Tmc retains TmcA within its gene neighborhood and trees of TmcA, TmcB, TmcC and TmcD also show that Tmc within HotSeep-1 is very similar to that of its nearest evolutionary neighbors Desulfofervidaceae (TmcB, TmcC trees are made available online https://github.com/ranjani-m/syntrophic-SRB). This is consistent with the fact that HotSeep-1 can use H_2_ as its electron donor in addition to using electrons from ANME-1 as its electron donor.

In addition to inference of adaptation from phylogenetic trees, our genomic data suggests that there are different electron donors in the periplasmic space of syntrophic SRB that are unique to syntrophic SRB besides TpI*c_3_*. This is corroborated by the high levels of expression of the periplasmic cytochromes *c* ExtA in Seep-SRB1a and ExtKL in Seep-SRB2 (**lines 342-344**, Krukenberg *et al.* Environmental Microbiology (2018), Yu *et al.* ISME 2021). These periplasmic cytochromes *c* are not present in other SRB and it is reasonable to expect that some complexes in syntrophic SRB are adapted to utilizing these cytochromes *c* as an electron donor. We are merely noting that it in Seep-SRB1a, this seems more likely to be Tmc rather than Qrc whose periplasmic cytochrome c subunit QrcA is not that different from those of the nearest evolutionary neighbors (available here <https://github.com/ranjani-m/syntrophic-SRB> and in online supplementary material). This does not have to change that energy conservation will involve Dsr, Qmo and Qrc.

To address your point that we have not made it clear that Tmc and Dsr will both likely reduce DsrC, we have made an addition to explicitly state that both DsrMKJOP and Tmc reduce DsrC. **Lines 367-370** “The respiratory pathways in HotSeep-1, Seep-SRB1a and Seep-SRB1g are broadly similar in structure and are predicted to use the Qrc complex to transfer periplasmic electrons to the quinone pool and both DsrMKJOP and Tmc to reduce cytoplasmic DsrC.”

We added a second sentence to support this assertion. **Lines 375-378.** “It is not clear why Tmc is more divergent than DsrMKJOP in syntrophic SRB compared to their evolutionary neighbors since they are both likely important for DsrC reduction and are equally well-expressed under methane oxidizing conditions[24,25].”

QmoC-fusion protein. It is now clarified that this protein is a fusion protein that has hemes c and a CCG domain. This suggests to me that it is not really "QmoC"-like, and so maybe a different name should be given to it. QmoC has an integral membrane domain with hemes b (not c), and a cytoplasmic domain with regular FeS clusters as present in HdrC. The protein described seems to be a fusion of a cytochrome c with a CCG protein (HdrB family) that contain a non-cubane cluster.

We agree that this protein could be given a more appropriate name. We have removed all mention of a QmoC-domain, and called it a putative cytochrome c oxidoreductase containing a CCG domain. The complex is described as follows **Lines 406 – 413**.

“HotSeep-1 genomes contain a complex that involves an HdrA subunit and a protein that also binds hemes c and contains a CCG domain similar to that found in HdrB and TmcB predicted to interact with DsrC[71,72]. This complex, a putative cytochrome c oxidoreductase containing a CCG domain, would likely transfer electrons from cytochromes *c* to the DsrC (AMM42179.1-AMM42180.1) or perhaps to a ferredoxin. The presence of HdrA might indicate a role in electron bifurcation by this complex. It is highly expressed during methane oxidation conditions in the ANME-1/HotSeep-1 consortia to a fifth of the level of the Tmc complex that would play a similar role in electron transfer[24].”

Lines 748-749: There certainly has to be a way for electrons from ANME to reach ferredoxin and NAD(P)+, but I doubt this necessarily involves DsrC. This protein is specific for sulfur metabolism, and although it may connect with the ferredoxin and NADH pools through Flx/Hdr, it is highly likely that other proteins will be involved in the direct reduction of Fd and NAD(P)+, which are likely conserved in other organisms capable of DIET, but not sulfur metabolism (Rnf, Nfn, and others).

One way for ferredoxin or NAD(P)+ to be reduced without the use of DsrC would require a complex that interacts with ferredoxin or NAD(P)+ and menaquinone or cytochrome c. We see no such complex in Seep-SRB1a, Seep-SRB1g or Seep-SRB2. Perhaps the above mentioned cytochrome *c* oxidoreductase containing a CCG domain could play such a role in HotSeep-1. The other way would be to directly expend proton motive force with the use Rnf to reduce ferredoxin. This might be possible but, given that there are multiple electron transfer complexes that likely reduce DsrC, it seems more likely as an electron donor. In any case, perhaps we could have framed our conclusions in a more cautious way. We have added a sentence to refer to different electron transfer pathways in **lines 431-437**. “The reduction of NADH, NADPH or ferredoxin could happen through the transfer of electrons from DsrC[76] or through the interconversion of electrons between cytoplasmic electron carriers through the dissipation of proton motive force or through the action of electron bifurcating complexes[77]. The transfer of electrons from DsrC to these reductants likely happens through the action of protein complexes like Flx-Hdr that can oxidize two molecules of NADH to reduce one molecule of ferredoxin and one molecule of DsrC. Electrons from NADH and ferredoxin can also be exchanged with the dissipation or generation of sodium motive force using membrane-bound Rnf and Mrp[77–79] in Seep-SRB1a, Seep-SRB1g and Hot-Seep1.”

I also think the manuscript would gain from some reduction and streamlining of the text.

We thank the reviewer for this suggestion. We appreciate that our manuscript is sometimes over-loaded with information and not always concise. However, the purpose of this manuscript was to provide a complex but detailed framework on which future studies can be based. We have attempted, to the best of our ability to re-read and streamline the text. In addition, we have added section headings to better organize the content and make it more readable. The comparative genome analysis section is now subdivided in this way –

Comparative genome analysis of syntrophic SRB

1. Respiratory pathways in the four syntrophic SRB clades demonstrate significant metabolic flexibility
   1.1 Multiple pathways exist for interspecies electron transfer between ANME and syntrophic SRB
   1.2 Pathways for electron transfer across the inner membrane vary in different syntrophic SRB clades
   1.3 Cytoplasmic redox reactions, electron bifurcation and carbon fixation pathways
2. Cobalamin auxotrophy and nutrient sharing in syntrophic SRB
3. Pathways related to biofilm formation and intercellular communication
   3.1 Multiple polysaccharide biosynthesis pathways are found in syntrophic SRB
   3.2 Several putative adhesins found in syntrophic SRB are absent in free-living SRB
   3.3 Secretion systems and intercellular communication in syntrophic SRB

Reviewer #2: The authors have done a good job responding to my comments, it is appreciated that they clarify now that their data indicates physiological potential rather than actual experiments.  As I pointed out in my first review, the genomic analyses of the syntrophic SRB are still interesting from an evolutionary perspective .  The manuscript should have an impact, since as the authors point out many of the "SEEP" SRB clades have been historically mis-cited in the literature as syntrophic partners and this paper now shows clearly which of the subclades are syntrophic.  The horizontal gene transfer analysis is also very interesting.  I am satisfied with the changes to the manuscript and now recommend publication.

We thank reviewer 2 for their insight on what is significant within our manuscript and their recommendation.
